# Ultra-broadband, wide-angle plus-shape slotted metamaterial solar absorber design with absorption forecasting using machine learning

**DOI:** 10.1038/s41598-022-14509-y

**Published:** 2022-06-17

**Authors:** Shobhit K. Patel, Juveriya Parmar, Vijay Katkar

**Affiliations:** 1 Department of Computer Engineering, Marwadi University, Rajkot, Gujarat India; 2 Department of Electronics and Communication Engineering, Marwadi University, Rajkot, Gujarat India

**Keywords:** Metamaterials, Optical sensors, Solar energy and photovoltaic technology

## Abstract

Energy utilization is increasing day by day and there is a need for highly efficient renewable energy sources. Solar absorbers with high efficiency can be used to meet these growing energy demands by transforming solar energy into thermal energy. Solar absorber design with highly efficient and Ultra-broadband response covering visible, ultraviolet, and near-infrared spectrum is proposed in this paper. The absorption response is observed for three metamaterial designs (plus-shape slotted design, plus-shape design, and square-shape design) and one optimized design is used for solar absorber design based on its high efficiency. The design results are compared with AM 1.5 spectral irradiance response. The electric field response of the plus-shape slotted metamaterial design is also presented which matches well with the absorption results of different solar spectrum regions. The results proved that the attained absorption response showing wide angle of incidence. Machine learning is also used to examine the design data in order to forecast absorption for various substrate thickness, metasurface thickness, and incidence angles. Regression and forecasting simulations based on machine learning are used to try to anticipate absorber behaviour at forthcoming and intermediate wavelengths. Simulation results prove that Machine Learning based methods can lessen the obligatory simulation resources, time and can be used as an effective tool while designing the absorber. The proposed highly efficient, wide-angle, ultra-broadband solar absorber design with its behavior prediction capability using machine learning can be utilized for solar thermal energy harvesting applications.

## Introduction

With the advancement in science and technology and rapid decrease in the cost of electrical, electronic equipment, energy demand is increased rapidly over the period. It is very difficult and not advisable to meet this requirement using conventional energy production methods. Renewable energy is a good solution to this problem as it will not hinder the environment and its ecosystem. Sun provides a gargantuan amount of light and heat energy to earth which can be used to generate clean renewable energy and satisfy the increased energy demand. Three approaches for solar energy harvesting are: (a) Photovoltaic approach: Use the photovoltaic device to convert photon energy into electricity (b) Photochemical approach: Convert solar energy into storable chemical fuel (c) Photothermal approach: Use solar thermal absorber to convert photon energy into thermal energy.

Solar absorbers are designed using conventional materials like Au, Ti-Al_2_O_3_ that cannot attain tunable performance. However, the use of carbon-based materials provides benefits like admirable thermal and chemical stability, first-rate thermal conductivity^1,2^. For the first time in the history of science, graphene has been discovered to be an atom-thick, 2D) carbon substance with exceptional electrical, crystallographic, and optical properties^3^. One layer of graphene is able to absorb 2.3 percent of white light with 0.1 percent refractivity^4^. It is further demonstrated that as the number of layers of graphene grows, so does the absorption, demonstrating the linear relationship^5^. As result graphene is used by many researchers for designing the solar absorber^6–9^.

Metamaterials is another excellent artificial material which can be used in designing absorber, sensors, etc. Metamaterials, which are purposefully constructed structures, have attracted a lot of consideration due to their outstanding qualities, such as symmetric transmission^10^, and negative refractive index^11^, and so on. The first MA (metamaterial absorber) was created by Landy et al.^12^, which achieved nearly complete absorption. The initial layer of these absorbers had a patterned metallic design to provide impedance matching and reduce light reflection. Using a resonant cavity design, the second dielectric layer allows electromagnetic waves to disperse before reaching the metallic plate layer, which stops transmission^13^. Absorption response is narrowband, with either single, double or, multiple peaks whose absorption response is unity^14–18^ and this is the only drawback of these absorbers. Solar absorbers must be very efficient in all wavelength ranges, including terahertz, ultraviolet, visible infrared and microwave^19–21^. Solar absorbers are used in a variety of applications, including solar energy harvesting, superlenses, and optical structure design^22–24^. The use of machine learning approaches like regression analysis, neural networks for pattern and behavior prediction is an active area of research in recent times^25–27^ and this techniques has also been applied for photonics devices in^28–31^. Regression analysis is a statistical approach in which a relationship between a dependent variable (in our case absorption value) and an independent variable (in our case wavelength value) is discovered. Time series analysis is used to discover the pattern of change in data values over a time period (in our case change in values of absorption over a change in wavelength values) under assorted conditions.

In this paper, we have proposed three metamaterial solar absorbers for the ultra-broadband range that includes ultraviolet, visible, and near-infrared. Section II contains the design, modeling, and results of these designs. The absorption analysis, graphene conductivity model, and shape analysis are also included in this section. Section III includes the parametric analysis of the design and electric field analysis. Section IV includes the machine learning analysis. Concluding notes are reported in section V.

## Design, modelling, and results

**Figure 1:**
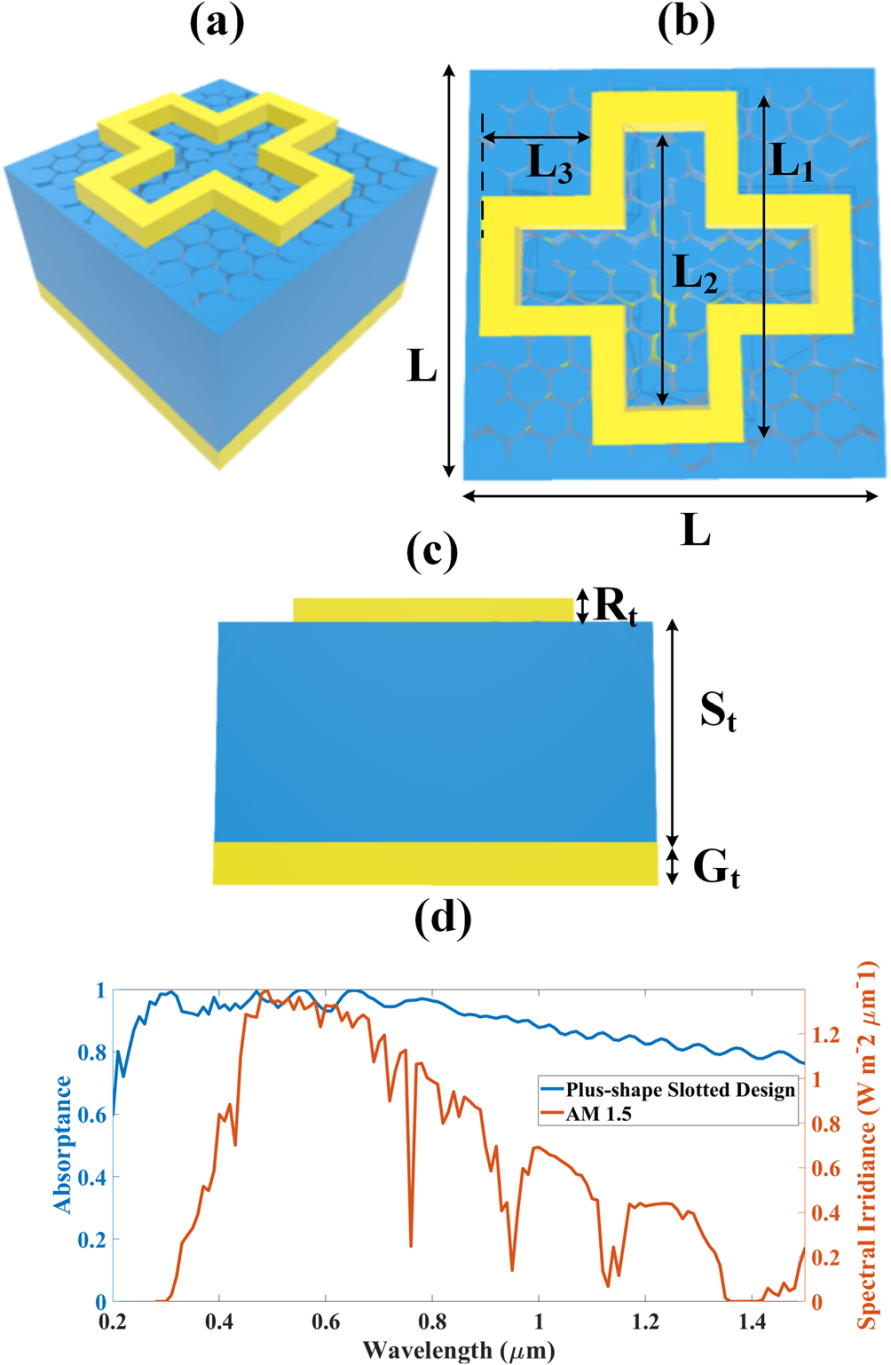
Plus-shape metamaterial-based solar absorber design (**a**) 3D view, (**b**) Top view, (**c**) Front view, (**d**) Ultra-broadband absorption response of plus-shape slotted metamaterial-based solar absorber design with AM 1.5. The parameters are: L = 3 µm, L_1_ = 1.9 µm, L_2_ = 1.4 µm, L_3_ = 1 µm, S_t_ = 8 µm, G_t_ = 0.5 µm, R_t_ = 0.5 µm. The figure is not up to the scale.

The solar absorber design (Fig. 1) is made by placing a plus-shape slotted gold resonator layer over SiO_2_ substrate and separated by graphene spacer. The gold ground plane is placed at the bottom of the structure to avoid transmittance. The thickness of the metamaterial resonator and substrate is kept at 0.5 µm and 8 µm subsequently. The graphene monolayer is 0.34 nm thick. The other dimensions regarding the length and width of the plus layer are shown in Fig. 1a–c. The metamaterial solar absorber design is simulated using COMSOL Multiphysics simulator through Finite Element Method (FEM) and the absorption result is presented in Fig. 1d. The designing of the graphene layer and absorption analysis is numerically presented here. The plus-shape slotted metamaterial solar absorber attains 91%, 97%, and 86% average absorption in the ultraviolet, visible, and NIR region, subsequently giving the ultra-broadband absorption response as observed in Fig. 1d.

### Absorption analysis

The absorption analysis for different angles of incidence can be calculated based on the following Eqs. (1–8) which calculated the reflectance for different angles and from reflectance the absorption can be calculated as there is almost no transmittance because of the gold ground plane. The dependence of absorption is on graphene’s conductivity (σ) and angle of incidence (θ_i_), as presented in Eqs. (1–8)^32^. In these equations $\omega$ is the angular frequency, $\mathrm{\hslash }$ is reduced plank constant, $k$ *is the* wave vector

1 $$r\left(\omega ,{\theta }_{i}\right)=\frac{\mathrm{cos}\,{\theta }_{i}{\prod }_{00}\left(\omega ,{\theta }_{i}\right)}{2ic{k}^{2}+\omega \mathrm{cos}\,{\theta }_{i}{\prod }_{00}\left(\omega ,{\theta }_{i}\right)}$$

2 $${\sigma }_{||}\left(\omega ,k\right)= X=-i \frac{\omega }{4\pi \hslash {k}^{2}}{\prod }_{00}\left(\omega ,k\right)$$

3 $$r\left(\omega ,{\theta }_{i}\right)=\frac{2\pi \mathrm{cos}\,{\theta }_{i}{\sigma }_{||}\left(\omega ,k\right)}{c+2\pi \mathrm{cos}\,{\theta }_{i}{\sigma }_{||}\left(\omega ,k\right)}$$

4 $$\mathcal{R}\left(\omega ,{\theta }_{i}\right)={\left|r\left(\omega ,{\theta }_{i}\right)\right|}^{2}$$

5 $$\mathcal{R}\left(\omega ,{\theta }_{i}\right)= \frac{4{\pi }^{2}{\mathit{cos}}^{2}\,{\theta }_{i}\left[{\mathrm{Re}}^{2}X+{\mathrm{Im}}^{2}X\right]}{{\left[c+2\pi \mathit{cos}\,{\theta }_{i}\mathrm{Re}X\right]}^{2}+4{\pi }^{2}{\mathit{cos}}^{2}\,{\theta }_{i}{\mathrm{Im}}^{2}X}$$

6 $$\mathcal{R}\left(\omega \right)=\mathcal{R}\left(\omega ,0\right)=\frac{4{\pi }^{2}\left[{\mathrm{Re}}^{2}\sigma \left(\omega \right)+{\mathrm{Im}}^{2}\sigma \left(\omega \right)\right]}{{\left[c+2\pi \mathrm{Re}\sigma \left(\omega \right)\right]}^{2}+4{\pi }^{2}{\mathrm{Im}}^{2}\sigma \left(\omega \right)}$$

7 $$A\left(\omega \right)=1-\mathcal{R}\left(\omega )-T(\omega \right)$$

As already stated, the transmittance is considered zero because of the gold ground plane. The Eq. (7) now becomes.

8 $$A\left(\omega \right)=1-\mathcal{R}(\omega )$$

The graphene conductivity model is very important in getting high absorption and is presented here.

### Graphene conductivity model

The intramodal and intermodal conductivity equations are presented in Eqs. (9–12)^30,33,34^

9 $$\varepsilon \left(\omega \right)=1+\frac{{\sigma }_{s}}{{\varepsilon }_{0}\omega \Delta }$$

10 $${\sigma }_{intra}=\frac{-j{e}^{2}{k}_{B}T}{\pi {\hslash }^{2}(\omega -j2\Gamma )}\left(\frac{{\mu }_{c}}{{k}_{B}T}+2\mathit{ln}\left({e}^{-\frac{{\mu }_{c}}{{k}_{B}T}}+1\right)\right)$$

11 $${\sigma }_{inter}= \frac{-j{e}^{2}}{4\pi \hslash }\mathit{ln}\left(\frac{2\left|{\mu }_{c}\right|-(\omega -j2\Gamma )\hslash }{2\left|{\mu }_{c}\right|+(\omega -j2\Gamma )\hslash }\right)$$

12 $${\sigma }_{s}={\sigma }_{inter}+{\sigma }_{intra}$$

## Parametric analysis and electric field results

**Figure 2:**
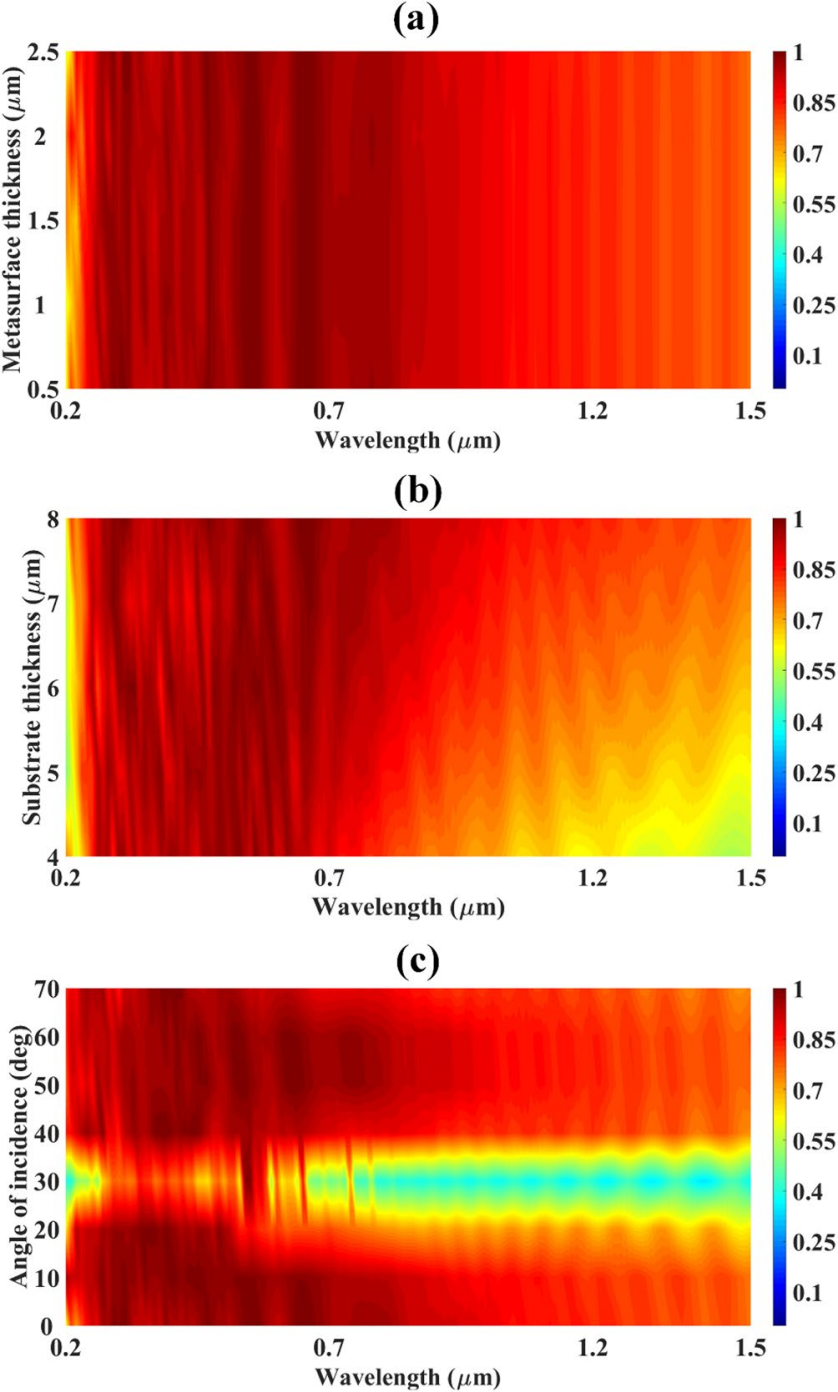
Absorption response of plus-shape slotted metamaterial solar absorber design while varying several physical parameters (**a**) Variation in absorption response while varying metasurface thickness. (**b**) Variation in absorption response while varying substrate thickness, (**c**) Absorption response while varying the angle of incidence.

The detailed analysis of the high-performing design of plus-shape slotted metamaterial-based solar absorber is carried out by varying various physical parameters such as metasurface thickness, substrate thickness, and angle of incidence. The corresponding results are presented in Fig. 2a–c.

Figure 2a illustrates the variation in absorption response with respect to variation in metasurface thickness. The metasurface thickness is varied from 0.5 to 2.5 µm with a step increase of 0.5 µm. It is visible that the absorption response is not very much affected by this change. So, we can deduce that to make the solar absorber cost-effective, the metasurface thickness is kept at 0.5 µm. Figure 2b illustrates the variation in absorption response w.r.t to increment in substrate thickness. It is observed that the absorption starts to increase as we increase the substrate thickness. The absorption response is excellent in the wavelength region of 0.2 µm to 1 µm. But for lower values of substrate thickness, we attain less absorption in the wavelength span of 1 µm to 1.5 µm as we can observe in Fig. 2b. But the absorption response in this particular area starts to increase as the substrate thickness rises to 8 µm. So, to achieve the high absorption, efficient, and ultra-broadband solar absorber we kept the substrate thickness at 8 µm. Figure 2c proves the wide-angle, angle insensitivity features of the proposed plus-shape slotted metamaterial solar absorber. As it is reported in Fig. 2c, the absorption response is identical for most of the angles except for the angle of incidence of 30°. But still, for 30°, we achieve average absorption of about 60% for the whole region covering from 0.2 to 1.5 µm. And for the rest of the angle of incidence, the attained average absorption response is above 90%. So we can state that the proposed solar absorber is wide-angle sensitive for 0° to 70°.

**Figure 3:**
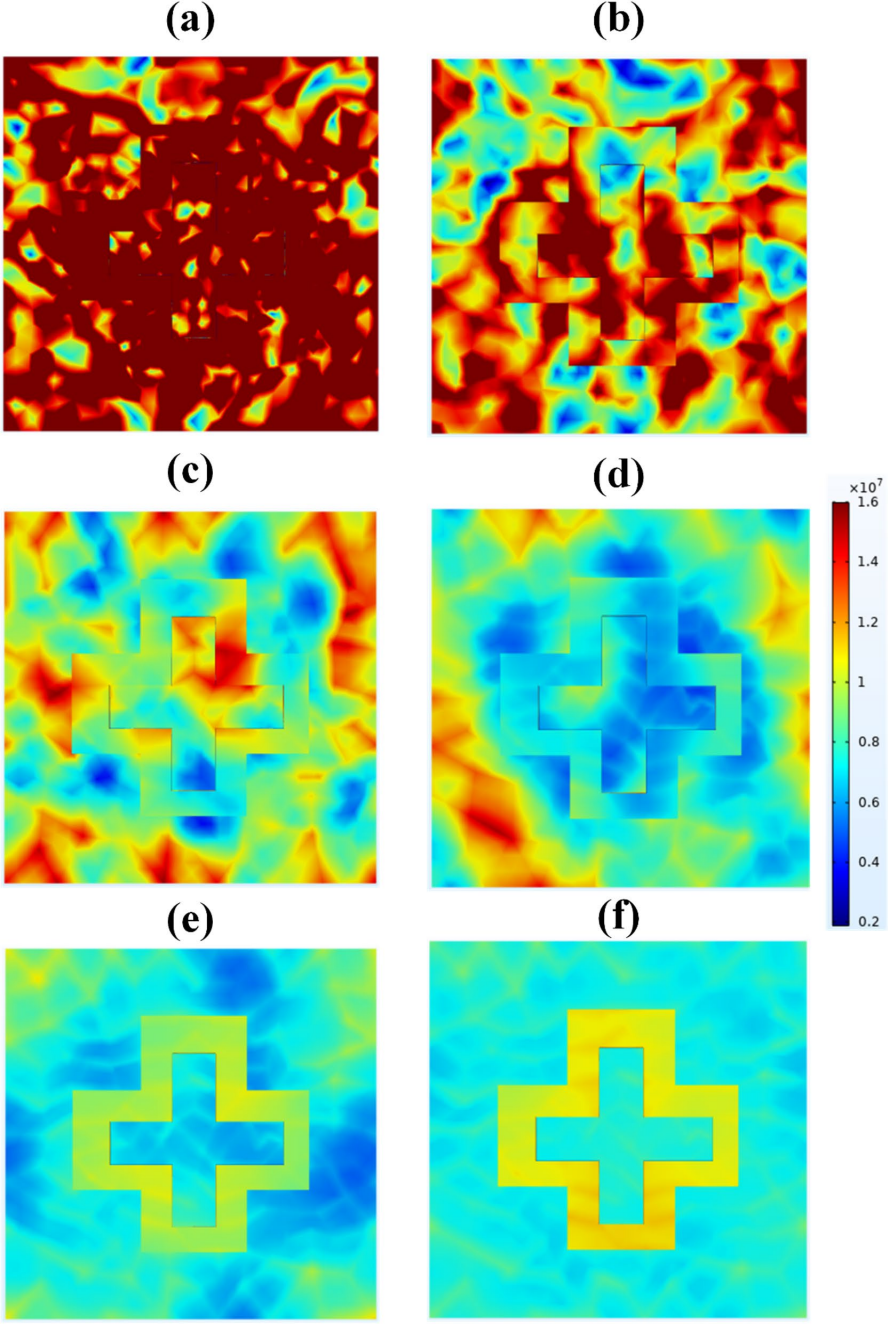
Electric field intensity of plus-shape slotted metamaterial solar absorber (**a**) 0.3 µm, (**b**) 0.5 µm, (**c**) 0.7 µm, (**d**) 0.9 µm, (**e**) 1.1 µm, (**f**) 1.3 µm.

The electric field intensity response of the proposed solar absorber is presented in Fig. 3 for various wavelength values so that it covers all the covered regions from 0.2 to 1.5 µm. The electric field response is attained for the wavelength values of 0.3 µm, 0.5 µm, 0.7 µm, 0.9 µm, 1.1 µm, and 1.3 µm. It represents that as we increase the wavelength the electric field intensity starts to decrease. The highest value of electric field intensity is achieved for 0.3 µm indicating the highest absorption that is of around 0.997 at that particular wavelength. So, the electric field intensity reported in Fig. 3 validates the absorption response achieved in Fig. 1d.

Furthermore, we have also compared our proposed solar absorbers with previously published work and the results are presented in the table format in Table 1.

**Table 1:** Comparison of the developed solar absorber with the available structures.

Design	Average absorption (%)			Angle insensitive
	Ultraviolet	Visible	Near-infrared	
Plus-shape slotted metamaterial design	91	97	86	0° to 70°
Ref.^37^	–	71.1	–	0° to 40°
Ref.^38^	92.99	86.5	86.1	–
Ref.^39^	–	–	90%	–
Ref.^40^	–	Above 90%	Above 90%	–
Ref.^41^	–	92	–	0° to 90°
Ref.^42^	–	70	–	–
Ref.^43^	–	80	–	–
Ref.^44^	89.57	97.51	85.48	–
Ref.^45^	74.1	92.1	–	0° to 30°

## Machine learning analysis

Weighted KNN-Regression is one of the widely used approaches for discovering the association amid independent and dependent variables^35^. The proficiency of a regression model is determined using a metric R^2^ score. This metric is calculated using Eq. (13).

13 $${R}^{2}= 1- \frac{{SS}_{red}}{{SS}_{tot}}$$

14 $${SS}_{tot}=\sum_{i=1}^{N}{({Actual \, Target}_{i}- Average \, Target \, Value )}^{2}$$

15 $${SS}_{res}={\sum }_{1}^{N}{({Predicted \, Target \, Value}_{i}-{Actual \, Target \, Value}_{i})}^{2}$$

Here, actual target value is actual absorption value, and predicted target value refers to anticipated absorption capacity by regression model after training.

### Weighted KNN-regression

Working of Weighted KNN-Regression is explained with the help of following algorithm.

**Algorithm: **Weighted KNN-Regression.

**Input:**

TD: Training Data.

K: Value of ‘K’.

x: Value of the independent variable for prediction.

**Procedure:**

Step 1: Compute distances amid ‘x’ and value of independent variable for every d ∈ TD.

Step 2: Select top ‘K’ items from TD with smallest distance from ‘x’.

Step 3: Compute predicted value using Eq. (16)

16 $$Predicted Value= \frac{{\sum }_{i=1}^{K}{w}_{i}*f({d}_{i})}{{\sum }_{i=1}^{K}{w}_{i}}$$

where $${w}_{i}= \frac{1}{d{istance(x, {d}_{i})}^{2}}$$

Here, *f*(d_i_), gives the value of dependent variable corresponding to training sample d_i_. Euclidian distance is most widely used distance measure.

### Time series analysis

It is used to discover the pattern of change in data values over a time period (in our case change in values of absorption over a change in wavelength values) under assorted conditions. This established pattern is then utilised to forecast future values (in our case absorption for forthcoming wavelengths). Mean Absolute Percentage Error (MAPE) is utilised to quantify the precision of time series models. Method to calculate MAPE is shown in Eq. (17):

17 $$MAPE=\frac{1}{n}\sum_{i=1}^{n} \left\lfloor {\frac{{A}_{i}-{F}_{i}}{Ai}} \right\rfloor  $$

where, F_i_, A_i_ is Forecasted values of absorption and Actual/Simulated values of absorption, and Count of forecasted values by model after training is ‘n’.

Time Series analysis model built using Long Short Term Memory (LSTM) is Neural Network based model for forecasting^36^ which can reminisce the values from preceding stage of Neural Network. Supplementary Fig. S1 depicts the memory cell unit of LSTM. It has self-connections to remember the state of memory cells based on time and multiplicative units (gates) to regulate how information flows. Input gate and output gate control the stream of input activation, cell activation subsequently. The internal state of the cell is scaled by forget gate and is added back to the cell as input through a self-recurrent connection. In supplementary Fig. S1, x_t_ shows the input to a memory cell at time t. c_t_ and c_t-1_ show the next cell state and the earlier cell state, correspondingly. h_t_, h_t-1_ stand for the hidden layer’s output at times t and t − 1.

Predicting future values or missing interval values is possible with regression analysis. It has a high degree of accuracy in predicting the values of missing intervals. Time series analysis, on the other hand, is more accurate at predicting future values than regression analysis. Simulators can save time and resources by employing the following strategy during runtime:

- **Step 1:** Perform Simulation using higher step size for values of wavelength.
- **Step 2:** Train Regression and Time Series model using simulated data.
- **Step 3:** Use regression analysis to envisage values of absorption of in-between/ intermediate wavelengths (which are omitted during the simulation process).
- **Step 4:** Use the Time Series model to envisage values of absorption for forthcoming wavelengths.

Regression analysis and time series analysis can be used to figure out what the absorption values will be for intermediate wavelength and forthcoming wavelength subsequently after performing the simulation on limited wavelength values in assorted conditions. Here time and resource requirements for simulation are reduced as the simulation is performed for fewer wavelength values.

### Simulation using machine learning

Python 3.8 and the scikit-learn library version 0.23.1 are used to implement weighted KNN-regressor models. LSTM model for time series analysis is implemented and trained using python 3.8 and tensorflow library version 2.4.

### Intermediate wavelength’s absorption value prediction using regression analysis

Four Test Cases (TCs), Test-0.6, Test-0.5, Test-0.4, and Test-0.3, are made to find out how much simulation time and resources can be cut. In test case TC-M, uniform random sampling is used to randomly select (1 − M) × 100 percent records from simulation data to train the regression analysis model, and remaining M × 100% simulated data points are used to test regression model's prediction effectiveness after training.

**Figure 4:**
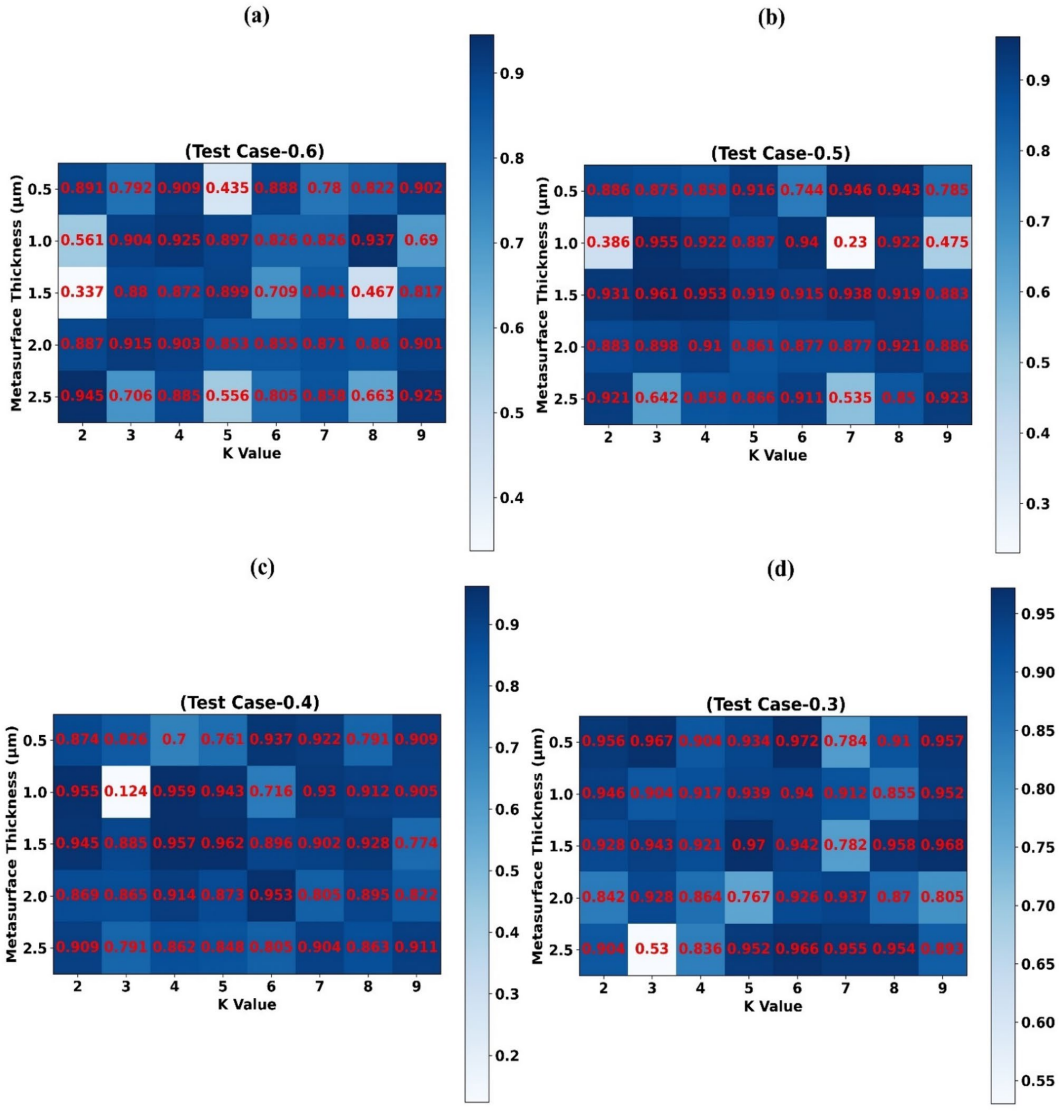
Prediction effectiveness (R2 Score) of traine Weighted KNN-regressor models for numerous values of Metasurface Thickness (µm) and (**a**) Test-0.6 (**b**) Test-0.5 (**c**) Test-0.4 (**d**) Test-0.3

Prediction effectiveness (R^2^ Score) of trained weighted KNN-regressor models for numerous values of metasurface thickness and Test-0.6, Test-0.5, Test-0.4, Test-0.3 is depicted in Fig. 4a–d subsequently with the help of heat map. A heat map demonstrates that howbeit only half of the simulation data points are used to train the weighted KNN-regressor model (Test-0.5), the model is still able to accurately (R^2^ score > 0.9) estimates the values of absorption for the other half of the wavelength values.

**Figure 5:**
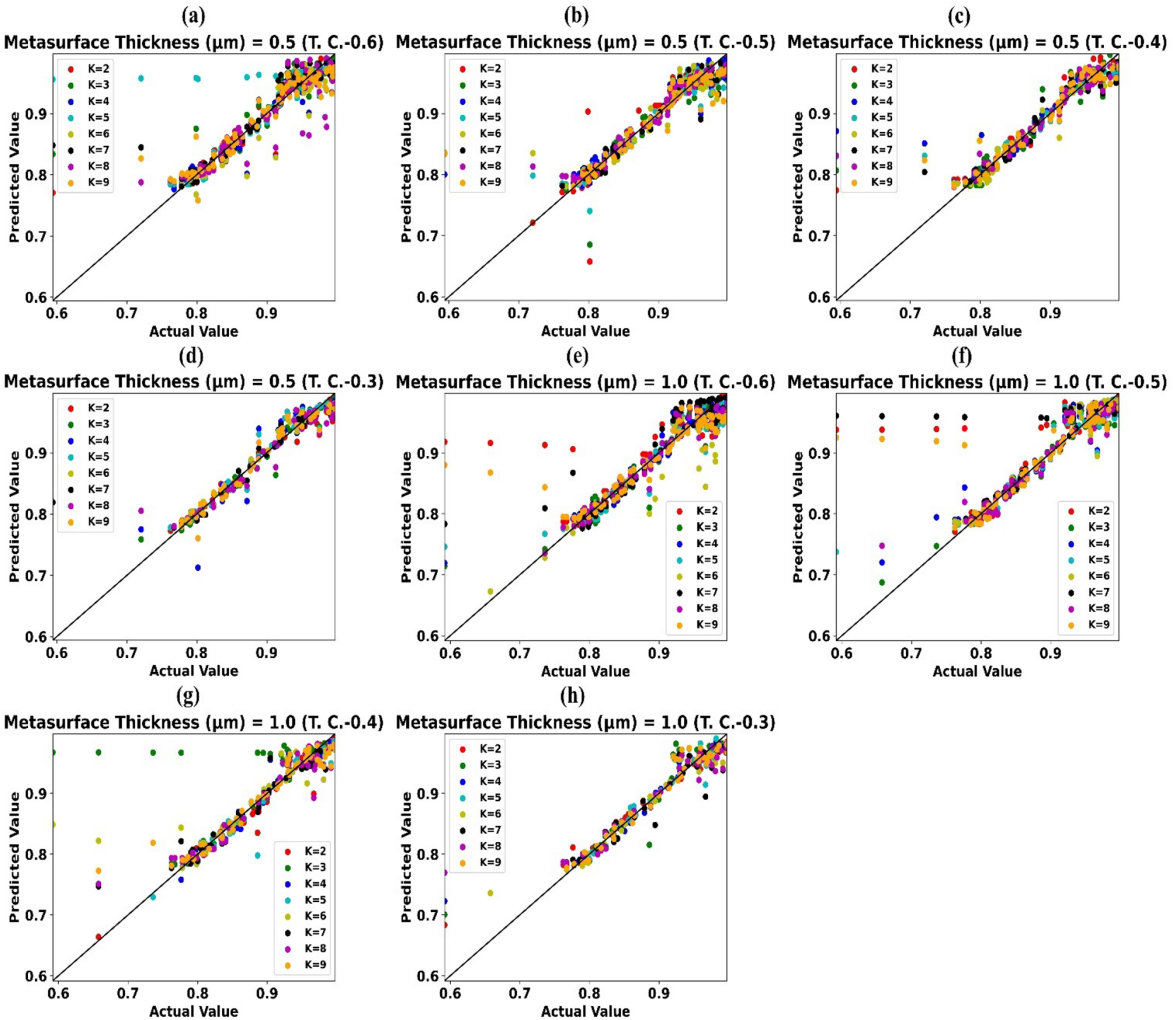
Predicted values of absorption by trained weighted KNN-regressor vs simulated/actual values of absorption for Metasurface Thickness (µm) (**a**) 0.5 (TC-0.6), (**b**) 0.5 (TC-0.5), (**c**) 0.5 (TC-0.4), (**d**) 0.5 (TC-0.3), (**e**) 1.0 (TC-0.6), (**f**) 1.0 (TC-0.5), (**g**) 1.0 (TC-0.4), (**h**) 1.0 (TC-0.3).

Scattergrams of estimated values of absorption by trained weighted KNN-regressor model vs simulated/actual values of absorption accomplished at the time of simulation for thickness of metasurface 0.5 µm and Test-0.6, Test-0.5, Test-0.4, Test-0.3 are revealed in Fig. 5a–d subsequently. Similarly, for thickness of metasurface 1.0 µm and Test-0.6, Test-0.5, Test-0.4, Test-0.3 are revealed in Fig. 5e–h subsequently. Results portrayed in Figs. 4 and 5 attests that trained weighted KNN-regressor model could cut the simulation resource and time requirement by half in this test situation.

Scattergrams of estimated values of absorption by trained weighted KNN-regressor model vs simulated/actual values of absorption accomplished at the time of simulation for thickness of metasurface 1.5 µm, 2 µm and Test-0.6, Test-0.5, Test-0.4, Test-0.3 are revealed in supplementary Fig. S2a–h subsequently.

Prediction effectiveness (R^2^ Score) of trained weighted KNN-regressor models for numerous values of angle of incidence and Test-0.6, Test-0.5, Test-0.4, Test-0.3 is depicted in supplementary Fig. S3a–d subsequently with the help of heat map. A heat map demonstrates that even though only 60% of the simulation records are used to train the weighted KNN-regressor model (Test-0.6), the model is still able to accurately (R^2^ score > 0.9) estimate the values of absorption for the outstanding wavelength values.

Scattergrams of estimated values of absorption by trained weighted KNN-regressor model vs simulated/actual values of absorption accomplished at the time of simulation for Incidence angle 0° and Test-0.6, Test-0.5, Test-0.4, Test-0.3 are revealed in supplementary Fig. S4a–d subsequently. Similarly, for incidence angle of 10° and Test-0.6, Test-0.5, Test-0.4, Test-0.3 are revealed in supplementary Fig. S4e–h subsequently. Results portrayed in Supplementary Figs. S3 and S4 attests that trained weighted KNN-regressor model could cut the simulation resource and time requirement by 60% in this test situation.

Scattergrams of estimated values of absorption by trained weighted KNN-regressor model vs simulated/actual values of absorption accomplished at the time of simulation for incidence angle 20°, 30° and Test-0.6, Test-0.5, Test-0.4, Test-0.3 are revealed in supplementary Fig. S5a–h subsequently. Scattergrams of estimated values of absorption by trained weighted KNN-regressor model vs simulated/actual values of absorption accomplished at the time of simulation for angle of incidence 40°, 50° and Test-0.6, Test-0.5, Test-0.4, Test-0.3 are revealed in Supplementary Fig. S6a–h subsequently. Scattergrams of estimated values of absorption by trained weighted KNN-regressor model vs simulated/actual values of absorption accomplished at the time of simulation for incidence angle 60°, 70° and Test-0.6, Test-0.5, Test-0.4, Test-0.3 are revealed in Supplementary Fig. S7a–h subsequently.

Prediction effectiveness (R^2^ Score) of trained weighted KNN-regressor models for numerous values of substrate thickness and Test-0.6, Test-0.5, Test-0.4, Test-0.3 is depicted in supplementary Fig. S8a–d subsequently with the help of heat map. A heat map demonstrates that howbeit only half of the simulation data points are used to train the weighted KNN-regressor model (TC-0.5), the model is still able to accurately (R^2^ score > 0.9) estimate the values of absorption for the other half of the wavelength values.

Scattergrams of estimated values of absorption by trained weighted KNN-regressor model vs simulated/actual values of absorption accomplished at the time of simulation for thickness of substrate 4 µm and Test-0.6, Test-0.5, Test-0.4, Test-0.3 are revealed in Supplementary Fig. S9a–d subsequently. Similarly, for thickness of substrate 5 µm and Test-0.6, Test-0.5, Test-0.4, Test-0.3 are revealed in Supplementary Fig. S9e–h subsequently. Results portrayed in supplementary Fig. S8 and S9 attests that trained weighted KNN-regressor model could cut the simulation resource and time requirement by half in this test situation.

Scattergrams of estimated values of absorption by trained weighted KNN-regressor model vs simulated/actual values of absorption accomplished at the time of simulation for thickness of substrate 6 µm, 7 µm and Test-0.6, Test-0.5, Test-0.4, Test-0.3 are revealed in Supplementary Fig. S10a–h subsequently.

Scattergrams of estimated values of absorption by trained weighted KNN-regressor model vs simulated/actual values of absorption accomplished at the time of simulation for metasurface width 1.9 µm, 2.0 µm and Test-0.6, Test-0.5, Test-0.4, Test-0.3 are revealed in Supplementary Fig. S11a–h subsequently. Scattergrams of estimated values of absorption by trained weighted KNN-regressor model vs simulated/actual values of absorption accomplished at the time of simulation for metasurface width 2.1 µm, 2.2 µm and Test-0.6, Test-0.5, Test-0.4, Test-0.3 are revealed in Supplementary Fig. S12a–h subsequently. Scattergrams of estimated values of absorption by trained weighted KNN-regressor model vs simulated/actual values of absorption accomplished at the time of simulation for metasurface width 2.3 µm, 2.4 µm and Test-0.6, Test-0.5, Test-0.4, Test-0.3 are revealed in Supplementary Fig. S13a–h subsequently.

Scattergrams of estimated values of absorption by trained weighted KNN-regressor model vs simulated/actual values of absorption accomplished at the time of simulation for metasurface length 1.9 µm, 2.0 µm and Test-0.6, Test-0.5, Test-0.4, Test-0.3 are revealed in Supplementary Fig. S14a–h subsequently. Scattergrams of estimated values of absorption by trained weighted KNN-regressor model vs simulated/actual values of absorption accomplished at the time of simulation for metasurface length 2.1 µm, 2.2 µm and Test-0.6, Test-0.5, Test-0.4, Test-0.3 are revealed in Supplementary Fig. S15a–h subsequently. Scattergrams of estimated values of absorption by trained weighted KNN-regressor model vs simulated/actual values of absorption accomplished at the time of simulation for metasurface length 2.3 µm, 2.4 µm and Test-0.6, Test-0.5, Test-0.4, Test-0.3 are revealed in Supplementary Fig. S16a–h subsequently.

### Regression analysis for predicting the values of absorption of forthcoming wavelengths

The layer structure of neural network-based LSTM forecasting model is shown in Supplementary Fig. S17. It contains one LSTM layer with 10 neurons and has a dense connection with other neurons in the neural network. During simulations, initial 80% simulation records are used to train the LSTM based forecasting models, and remaining 20% records are used to test the prediction effectiveness of forecasting models. Simulations are performed using different lengths of preceding inputs (7–13) used to forecast the absorption value for forthcoming wavelengths.

**Figure 6:**
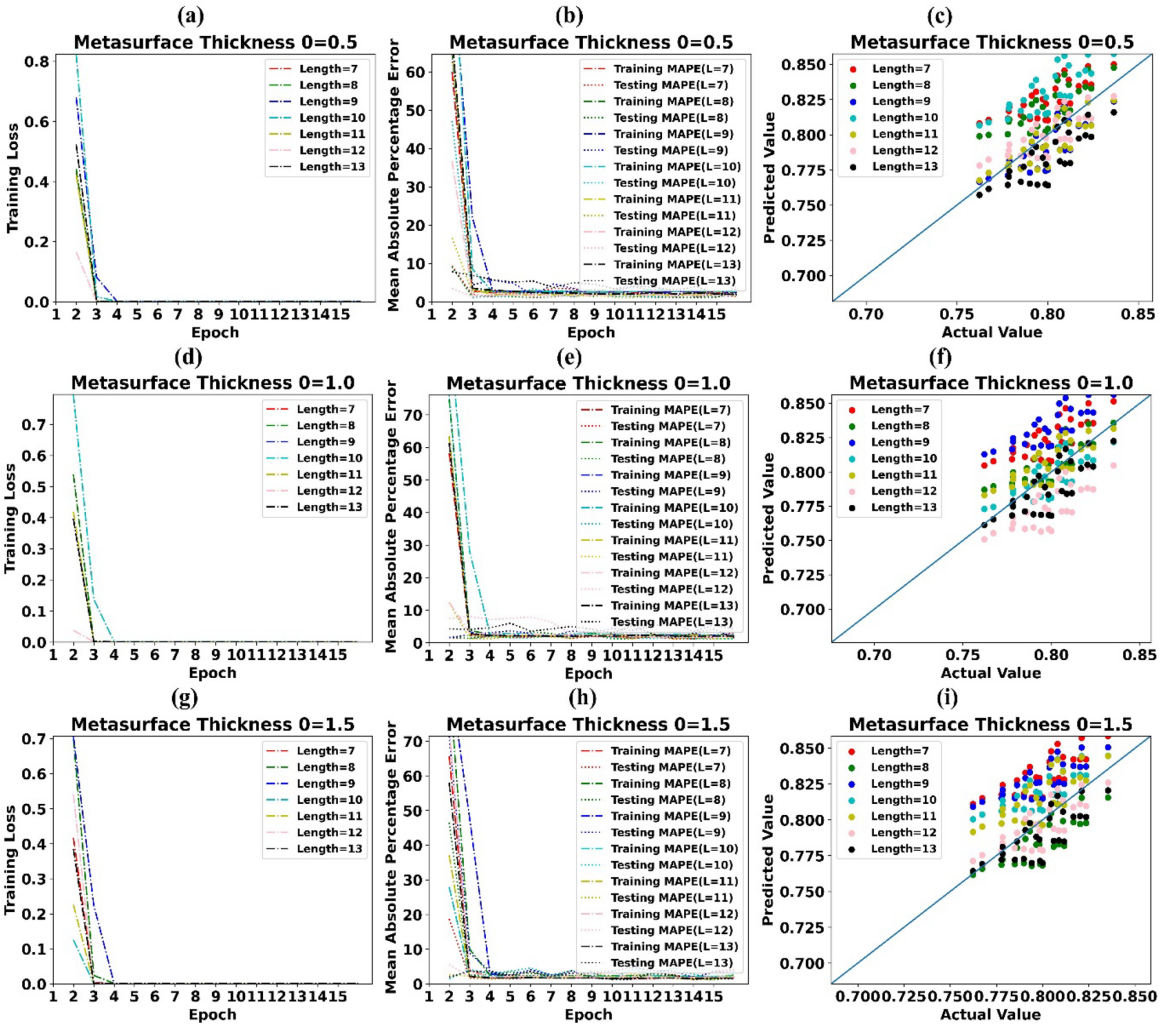
(**a**) Training Loss of LSTM model for Metasurface Thickness 0.5 µm (**b**) MAPE of LSTM model for Metasurface Thickness 0.5 µm (**c**) Predicted values of absorption by LSTM vs simulated values of absorption for Metasurface Thickness 0.5 µm (**d**) Training Loss of LSTM model for Metasurface Thickness 1.0 µm (**e**) MAPE of LSTM model for Metasurface Thickness 1.0 µm (**f**) Predicted values of absorption by LSTM vs simulated values of absorption for Metasurface Thickness 1.0 µm (**g**) Training Loss of LSTM model for Metasurface Thickness 1.5 µm (**h**) MAPE of LSTM model for Metasurface Thickness 1.5 µm (**i**) Predicted values of absorption by LSTM vs simulated values of absorption for Metasurface Thickness 1.5 µm.

Training loss, MAPE, and predicted values of absorption by LSTM vs simulated values of absorption for Metasurface thickness 0.5 µm is revealed in Fig. 6a–c subsequently. Likewise Fig. 6d–i depicts the alike information for Metasurface thickness 1.0 µm, 1.5 µm.

It can be simply detected from Fig. 6a,b,d,e,g,h that training loss reached zero after 4 training epoche and MAPE during the testing phase is around 1.0 percent. Scattergrams of predicted values of absorption by LSTM models vs simulated values of absorption in Fig. 6c,f,i show that, predicted values are very close to actual values of absorption. This in sequence supports that, forecasting models implemented using LSTM could cut the simulation resources and time by 20%.

Training loss, MAPE and predicted values of absorption by LSTM vs simulated values of absorption for metasurface thickness of 2 µm is shown in Supplementary Fig. S18a–c subsequently. Likewise Supplementary Fig. S18d–f depicts the alike information for angle of incidence 2.5 µm.

**Figure 7:**
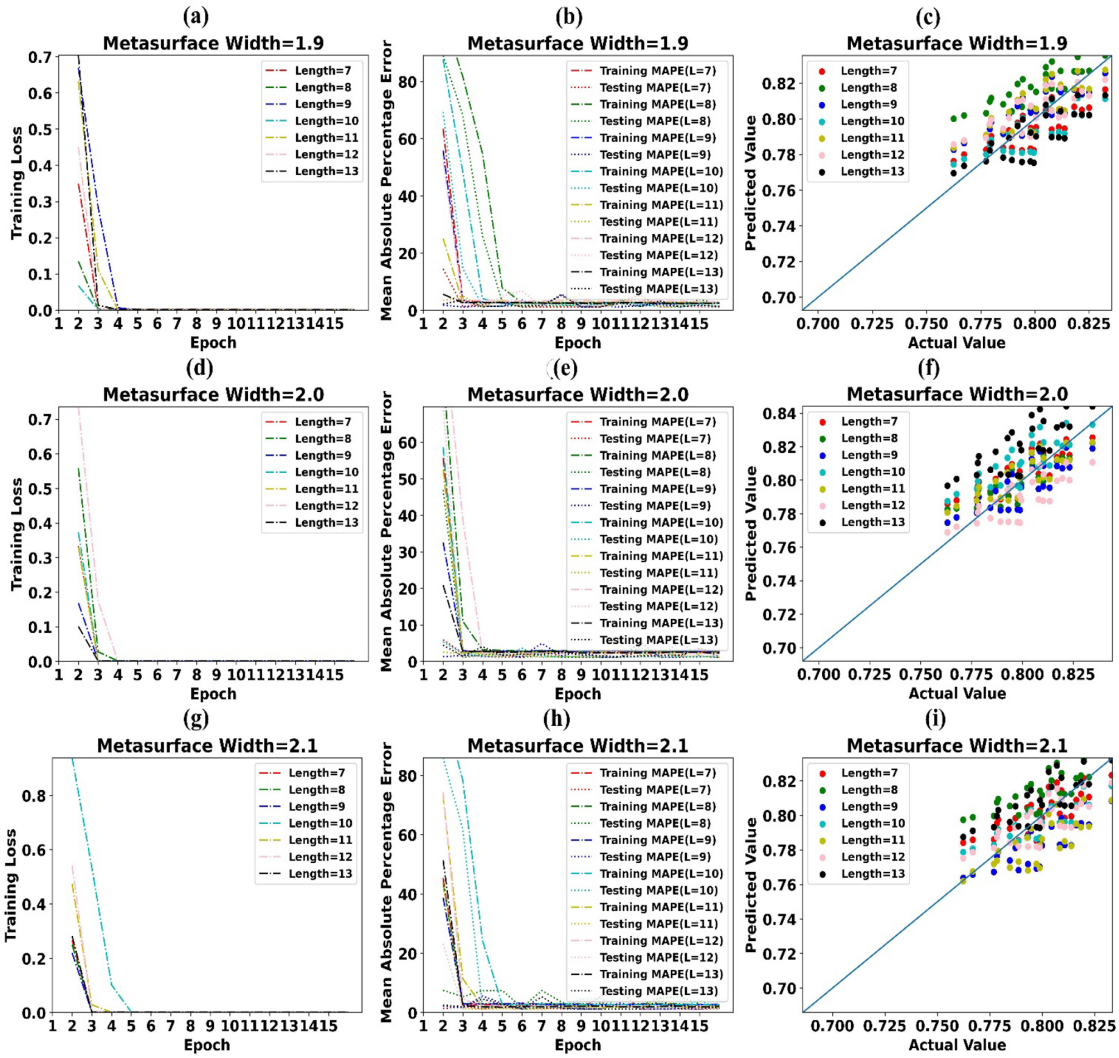
(**a**) Training Loss of LSTM model for Metasurface Width 1.9 µm (**b**) MAPE of LSTM model for Metasurface Width 1.9 µm (**c**) Predicted values of absorption by LSTM vs simulated values of absorption for Metasurface Width 1.9 µm (**d**) Training Loss of LSTM model for Metasurface Width 2.0 µm (**e**) MAPE of LSTM model for Metasurface Width 2.0 µm (**f**) Predicted values of absorption by LSTM vs simulated values of absorption for Metasurface Width 2.0 µm (**g**) Training Loss of LSTM model for Metasurface Width 2.1 µm (**h**) MAPE of LSTM model for Metasurface Width 2.1 µm (**i**) Predicted values of absorption by LSTM vs simulated values of absorption for Metasurface Width 2.1 µm.

Training loss, MAPE and predicted values of absorption by LSTM vs simulated/actual values of absorption for metasurface width of 1.9 µm is shown in Fig. 7a–c subsequently. Likewise, Fig. 7d–i depicts the alike information for metasurface width 2.0 µm and 2.1 µm. It can be simply detected from Fig. 7a,b,d,e,g,h that training loss reached zero after 4 training epoche and MAPE during the testing phase is around 1.0 percent. Scattergrams of predicted values of absorption by LSTM models vs simulated values of absorption in Fig. 7c,f,i show that, predicted values are very close to actual values of absorption. This in sequence supports that, LSTM based forecasting models could cut the simulation resources and time by 20%.

Training loss, MAPE and predicted values of absorption by LSTM vs simulated values of absorption for metasurface width of 2.2 µm is shown in Supplementary Fig. S19a–c subsequently. Likewise, Supplementary Fig. S19d–i depicts the alike information for metasurface width 2.3 µm and 2.4 µm.

Training loss, MAPE and predicted values of absorption by LSTM vs simulated values of absorption for metasurface length of 1.9 µm is shown in Supplementary Fig. S20a–c subsequently. Likewise, Supplementary Fig. S20d–i depicts the alike information for metasurface length 2.0 µm and 2.1 µm. It can be simply detected from Supplementary Fig. S20a,b,d,e,g,h that training loss reached zero after 4 training epoche and MAPE during the testing phase is around 1.0 percent. Scattergrams of predicted values of absorption by LSTM models vs simulated values of absorption in Supplementary Fig. S20c,f,i show that, predicted values are very close to actual values of absorption. This in sequence supports that, forecasting models implemented using LSTM could cut the simulation resources and time by 20%.

Training loss, MAPE and predicted values of absorption by LSTM vs simulated values of absorption for metasurface length of 2.2 µm is shown in Supplementary Fig. S21a–c subsequently. Likewise, Supplementary Fig. S21d–i depicts the alike information for metasurface length 2.3 µm and 2.4 µm.

Training loss, MAPE and predicted values of absorption by LSTM vs simulated values of absorption for Angle of Incidence 0° are shown in Supplementary Fig. S22a–c subsequently. Similarly, Supplementary Fig. S22d–i depicts the alike information for angle of incidence 10° and 20°.

It can be easily observed from Supplementary Fig. S22a,b,d,e,g,h that training loss reached zero after 5 training epoche and MAPE during the testing phase is around 1.0 percent. Scattergrams of predicted values of absorption by LSTM models vs simulated values of absorption in Supplementary Fig. S22c,f,i show that, predicted values are very close to actual values of absorption. This in sequence supports that, forecasting models implemented using LSTM could cut the simulation resources and time by 20% as initial 80% simulation records are used to train the LSTM based forecasting models, and remaining 20% records are predicted by models with high accuracy.

Training loss, MAPE and predicted values of absorption by LSTM vs simulated values of absorption for Angle of Incidence 30^0^ is shown in Supplementary Fig. S23a–c subsequently. Similarly Supplementary Fig. S23d–i depicts the alike information for angle of incidence 40°, 50° and Supplementary Fig. S24a–f depicts the alike information for angle of incidence 60°, 70°.

Training loss, MAPE, and predicted values of absorption by LSTM vs simulated values of absorption for substrate thickness 7 µm is shown in Supplementary Fig. S25a–c subsequently. Likewise, supplementary Fig. S25d–f depicts the alike information for substrate thickness 8 µm.

It can be easily observed from Supplementary Fig. S25a,b,d,e that training loss reached zero after 4 training epoche, and MAPE during the testing phase is around 1.0 percent. Scattergrams of predicted values of absorption by LSTM models vs simulated values of absorption in Supplementary Fig. S25c,f show that, predicted values are very close to actual values of absorption. This in sequence supports that, forecasting models implemented using LSTM could cut the simulation resources and time by 20%.

Training loss, MAPE and predicted values of absorption by LSTM vs simulated values of absorption for substrate thickness of 4 µm is shown in Supplementary Fig. S26a–c subsequently. Likewise Supplementary Fig. S26d–f depicts the alike information for substrate thickness of 5 µm. Training loss, MAPE and predicted values of absorption by LSTM vs simulated values of absorption for substrate thickness of 6 µm is shown in Supplementary Fig. S26g–i subsequently.

## Conclusion

Three metamaterial solar absorbers are analyzed for the ultra-broadband range of 0.2 µm to 1.5 µm including ultraviolet, visible, and near-infrared range. The highest average absorption of 91%, 97%, and 86% in the ultraviolet, visible, and near-infrared range for plus-shape slotted metamaterial solar absorber design is obtained. The overall average absorption of 90% is achieved. The shape of the metasurface is varied to check its effect on absorption response and the results show the absorption slightly reduces for the other two designs of plus-shape and square-shape metamaterial solar absorber designs. The detailed analysis of the best performing design is carried out by varying the physical parameters such as metasurface thickness, and substrate thickness to check its effect on absorption response. It is reported that the metasurface thickness has very less effect on absorption whereas the increment in substrate thickness increases the absorption response. The achieved absorption response is also angle insensitive for 0° to 70°. We can conclude that the proposed solar absorber is giving absorption response for ultra-broadband range and it is wide-angle. Simulations are performed using weighted KNN-regression and LSTM-based time series analysis model to predict the behavior of absorber for intermediate and forthcoming wavelength values. Simulation results illustrates that forecasting and regression models can predict the values of absorption with good efficiency/accuracy and could cut obligatory simulation resources, time, by 60% (40% by use of regression model and 20% by use of forecasting model).

## Supplementary Information

Supplementary Figures.

## Data Availability

The data will be made available at a reasonable request to the corresponding author.
