# Supplementary material for: Ultra-broadband, wide-angle plus-shape slotted metamaterial solar absorber design with absorption forecasting using machine learning
Source: Sci Rep. 2022 Jun 17;12:10166. doi: 10.1038/s41598-022-14509-y (PMC9206018; doi:10.1038/s41598-022-14509-y)
Supplement: Supplementary file 1 — Supplementary Figures. [file 41598_2022_14509_MOESM1_ESM.docx]

Supplementary Material

Supplementary Fig. S1 depicts the memory cell unit of LSTM.

Scattergrams of estimated values of absorption by trained weighted KNN-regressor model vs simulated/actual values of absorption accomplished at the time of simulation for thickness of metasurface 1.5µm, 2µm and Test-0.6, Test-0.5, Test-0.4, Test-0.3 are revealed in supplementary Fig. S2 (a-h) subsequently.

Prediction effectiveness (R^2^ Score) of trained weighted KNN-regressor models for numerous values of angle of incidence and Test-0.6, Test-0.5, Test-0.4, Test-0.3 is depicted in supplementary Fig. S3 (a-d) subsequently with the help of heat map.

Scattergrams of estimated values of absorption by trained weighted KNN-regressor model vs simulated/actual values of absorption accomplished at the time of simulation for Incidence angle 0^0^ and Test-0.6, Test-0.5, Test-0.4, Test-0.3 are revealed in supplementary Fig. S4 (a-d) subsequently. Similarly, for incidence angle of 10^0^ and Test-0.6, Test-0.5, Test-0.4, Test-0.3 are revealed in supplementary Fig. S4 (e-h) subsequently.

Scattergrams of estimated values of absorption by trained weighted KNN-regressor model vs simulated/actual values of absorption accomplished at the time of simulation for incidence angle 20^0^, 30^0^ and Test-0.6, Test-0.5, Test-0.4, Test-0.3 are revealed in supplementary Fig. S5 (a-h) subsequently.

Scattergrams of estimated values of absorption by trained weighted KNN-regressor model vs simulated/actual values of absorption accomplished at the time of simulation for angle of incidence 40^0^, 50^0^ and Test-0.6, Test-0.5, Test-0.4, Test-0.3 are revealed in supplementary Fig. S6 (a-h) subsequently.

Scattergrams of estimated values of absorption by trained weighted KNN-regressor model vs simulated/actual values of absorption accomplished at the time of simulation for incidence angle 60^0^, 70^0^ and Test-0.6, Test-0.5, Test-0.4, Test-0.3 are revealed in supplementary Fig. S7 (a-h) subsequently.

Prediction effectiveness (R^2^ Score) of trained weighted KNN-regressor models for numerous values of substrate thickness and Test-0.6, Test-0.5, Test-0.4, Test-0.3 is depicted in supplementary Fig. S8 (a-d) subsequently with the help of heat map.

Scattergrams of estimated values of absorption by trained weighted KNN-regressor model vs simulated/actual values of absorption accomplished at the time of simulation for thickness of substrate 4µm and Test-0.6, Test-0.5, Test-0.4, Test-0.3 are revealed in supplementary Fig. S9 (a-d) subsequently.

Scattergrams of estimated values of absorption by trained weighted KNN-regressor model vs simulated/actual values of absorption accomplished at the time of simulation for thickness of substrate 6µm, 7µm and Test-0.6, Test-0.5, Test-0.4, Test-0.3 are revealed in supplementary Fig. S10 (a-h) subsequently.

Scattergrams of estimated values of absorption by trained weighted KNN-regressor model vs simulated/actual values of absorption accomplished at the time of simulation for metasurface width 1.9µm, 2.0µm and Test-0.6, Test-0.5, Test-0.4, Test-0.3 are revealed in supplementary Fig. S11 (a-h) subsequently.

Scattergrams of estimated values of absorption by trained weighted KNN-regressor model vs simulated/actual values of absorption accomplished at the time of simulation for metasurface width 2.1µm, 2.2µm and Test-0.6, Test-0.5, Test-0.4, Test-0.3 are revealed in supplementary Fig. S12 (a-h) subsequently.

Scattergrams of estimated values of absorption by trained weighted KNN-regressor model vs simulated/actual values of absorption accomplished at the time of simulation for metasurface width 2.3µm, 2.4µm and Test-0.6, Test-0.5, Test-0.4, Test-0.3 are revealed in supplementary Fig. S13 (a-h) subsequently.

Scattergrams of estimated values of absorption by trained weighted KNN-regressor model vs simulated/actual values of absorption accomplished at the time of simulation for metasurface length 1.9µm, 2.0µm and Test-0.6, Test-0.5, Test-0.4, Test-0.3 are revealed in supplementary Fig. S14 (a-h) subsequently.

Scattergrams of estimated values of absorption by trained weighted KNN-regressor model vs simulated/actual values of absorption accomplished at the time of simulation for metasurface length 2.1µm, 2.2µm and Test-0.6, Test-0.5, Test-0.4, Test-0.3 are revealed in supplementary Fig. S15 (a-h) subsequently.

Scattergrams of estimated values of absorption by trained weighted KNN-regressor model vs simulated/actual values of absorption accomplished at the time of simulation for metasurface length 2.3µm, 2.4µm and Test-0.6, Test-0.5, Test-0.4, Test-0.3 are revealed in supplementary Fig. S16 (a-h) subsequently.

The layer structure of neural network-based LSTM forecasting model is shown in supplementary Fig. S17.

Training loss, MAPE and predicted values of absorption by LSTM vs simulated values of absorption for metasurface thickness of 2µm is shown in supplementary Fig. S18 (a-c) subsequently. Likewise supplementary Fig. S18 (d-f) depicts the alike information for angle of incidence 2.5 µm.

Training loss, MAPE and predicted values of absorption by LSTM vs simulated values of absorption for metasurface width of 2.2 µm is shown in supplementary Fig. S19 (a-c) subsequently. Likewise, supplementary Fig. S19 (d-i) depicts the alike information for metasurface width 2.3 µm and 2.4 µm.

Training loss, MAPE and predicted values of absorption by LSTM vs simulated values of absorption for metasurface length of 1.9 µm is shown in supplementary Fig. S20 (a-c) subsequently. Likewise, supplementary Fig. S20 (d-i) depicts the alike information for metasurface length 2.0 µm and 2.1 µm.

Scattergrams of predicted values of absorption by LSTM models vs simulated values of absorption in supplementary Fig. S20 (c, f, i) show that, predicted values are very close to actual values of absorption. This in sequence supports that, forecasting models implemented using LSTM could cut the simulation resources and time by 20%.

Training loss, MAPE and predicted values of absorption by LSTM vs simulated values of absorption for metasurface length of 2.2 µm is shown in supplementary Fig. S21 (a-c) subsequently. Likewise, supplementary Fig. S21 (d-i) depicts the alike information for metasurface length 2.3 µm and 2.4 µm.

Training loss, MAPE and predicted values of absorption by LSTM vs simulated values of absorption for Angle of Incidence 0^0^ are shown in supplementary Fig. S22 (a-c) subsequently. Similarly, supplementary Fig. S22 (d-i) depicts the alike information for angle of incidence 10^0^ and 20^0^.

Training loss, MAPE and predicted values of absorption by LSTM vs simulated values of absorption for Angle of Incidence 30^0^ is shown in supplementary Fig. S23 (a-c) subsequently. Similarly supplementary Fig. S23 (d-i) depicts the alike information for angle of incidence 40^0^, 50^0^ and supplementary Fig. S24 (a-f) depicts the alike information for angle of incidence 60^0^, 70^0^.

Training loss, MAPE, and predicted values of absorption by LSTM vs simulated values of absorption for substrate thickness 7µm is shown in supplementary Fig. S25 (a-c) subsequently. Likewise, supplementary Fig. S25 (d-f) depicts the alike information for substrate thickness 8 µm.

Training loss, MAPE and predicted values of absorption by LSTM vs simulated values of absorption for substrate thickness of 4µm is shown in supplementary Fig. S26 (a-c) subsequently. Likewise supplementary Fig. S26 (d-f) depicts the alike information for substrate thickness of 5 µm. Training loss, MAPE and predicted values of absorption by LSTM vs simulated values of absorption for substrate thickness of 6 µm is shown in supplementary Fig. S26 (g-i) subsequently.


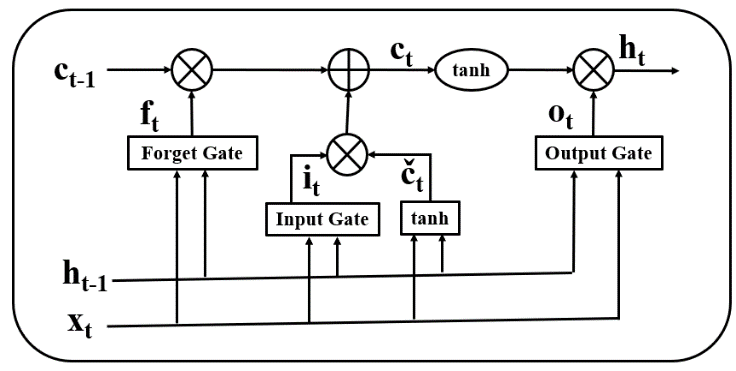


**Fig. S1** Memory Cell Unit of LSTM


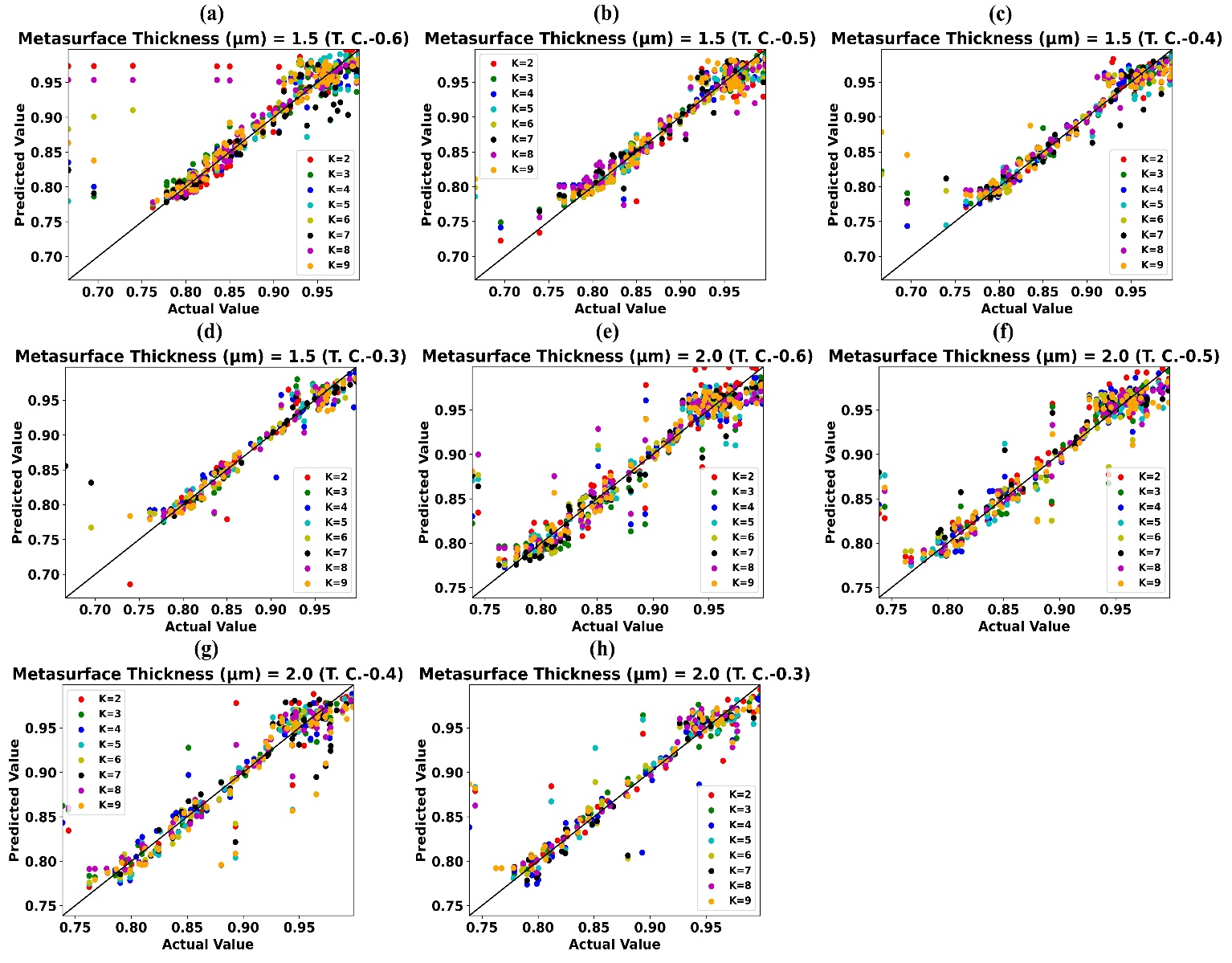


**Fig. S2** Predicted absorption value by weighted KNN-ressor vs actual absorption value for Metasurface Thickness (µm) (a) 1.5 (TC-0.6) (b) 1.5 (TC-0.5) (c) 1.5 (TC-0.4) (d) 1.5 (TC-0.3) (e) 2.0 (TC-0.6) (f) 2.0 (TC-0.5) (g) 2.0 (TC-0.4) (h) 2.0 (TC-0.3)


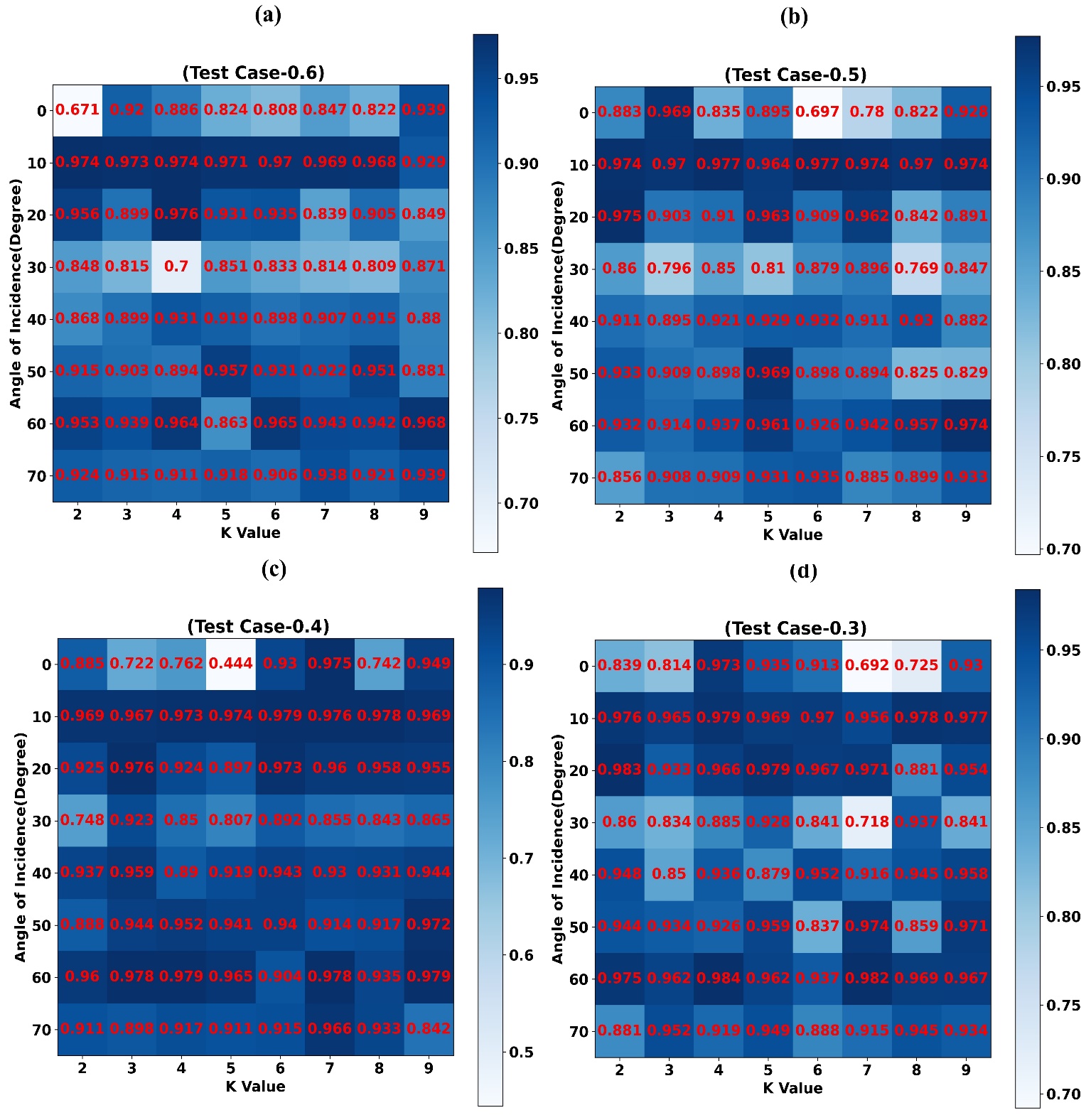


**Fig. S3** Prediction effectiveness (R2 Score) of Weighted KNN-regressor models trained for various values of Angle of Incidence (Degree) and (a) TC-0.6 (b) TC-0.5 (c) TC-0.4 (d) TC-0.3


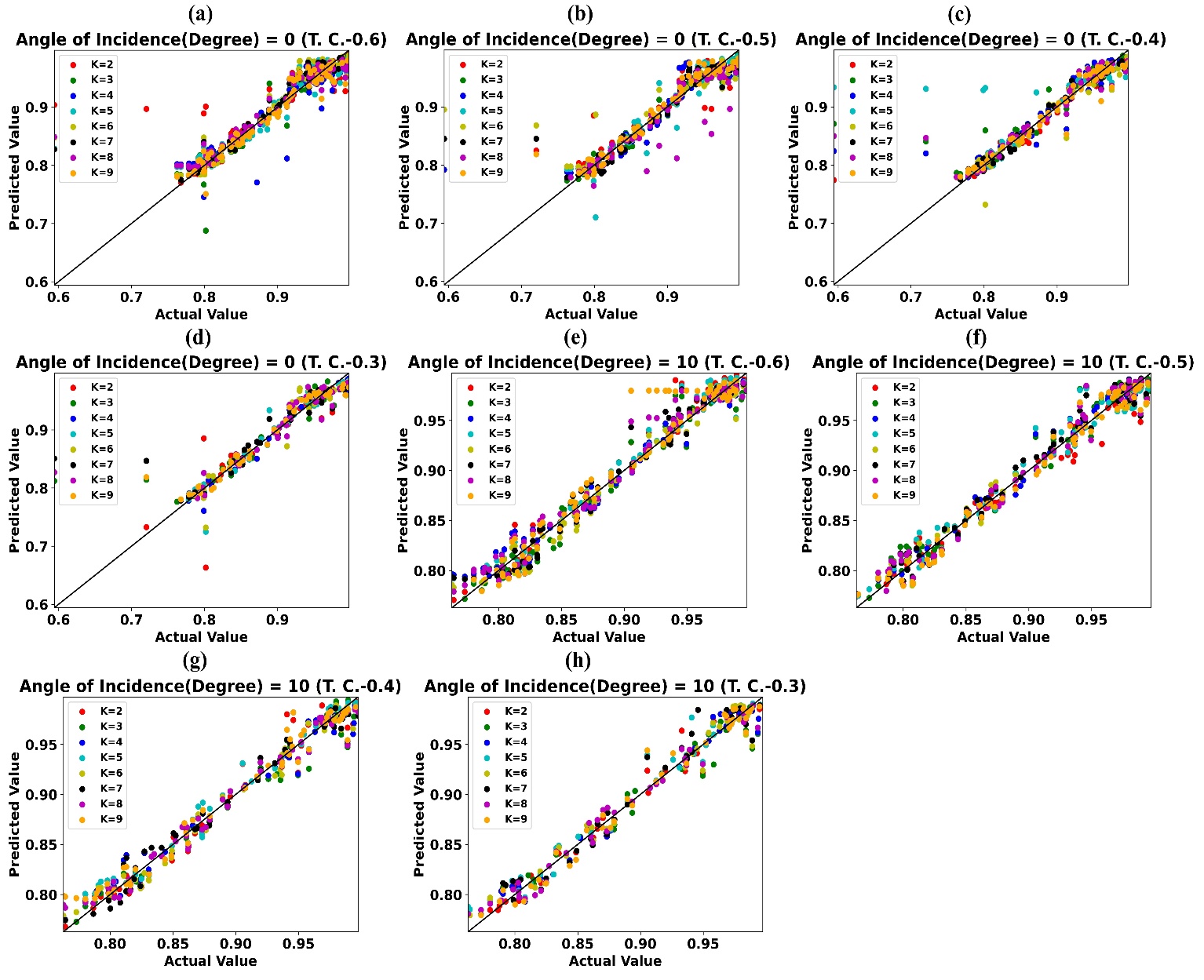


**Fig. S4** Predicted absorption value by weighted KNN-regressor vs actual absorption value for Angle of Incidence (degree) (a) 0^0^ (TC-0.6) (b) 0^0^ (TC-0.5) (c) 0^0^ (TC-0.4) (d) 0^0^ (TC-0.3) (e) 10^0^ (TC-0.6) (f) 10^0^ (TC-0.5) (g) 10^0^ (TC-0.4) (h) 10^0^ (TC-0.3)


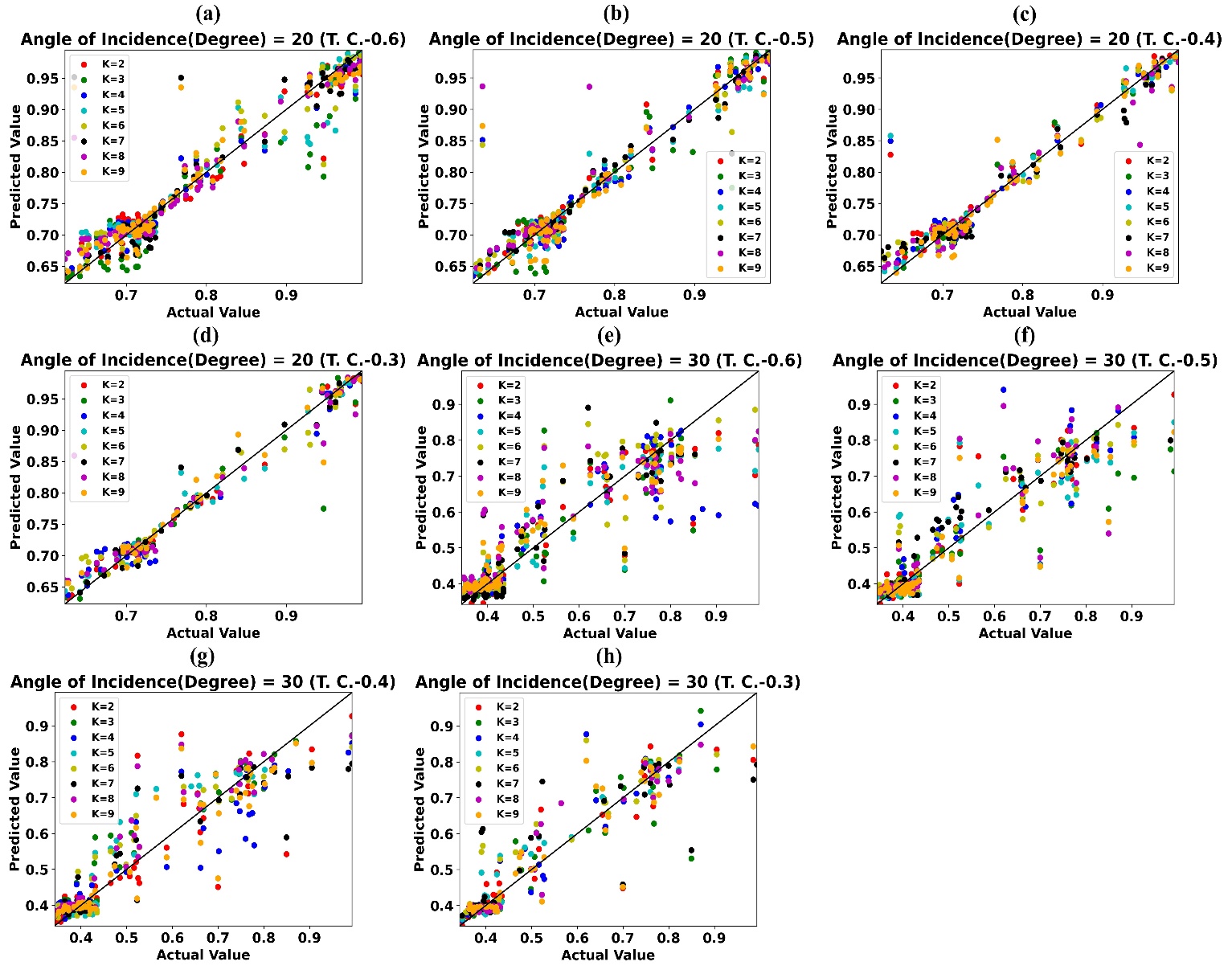


**Fig. S5** Predicted absorption value by weighted KNN-ressor vs actual absorption value for Angle of Incidence (degree) (a) 20^0^ (TC-0.6) (b) 20^0^ (TC-0.5) (c) 20^0^ (TC-0.4) (d) 20^0^ (TC-0.3) (e) 30^0^ (TC-0.6) (f) 30^0^ (TC-0.5) (g) 30^0^ (TC-0.4) (h) 30^0^ (TC-0.3)


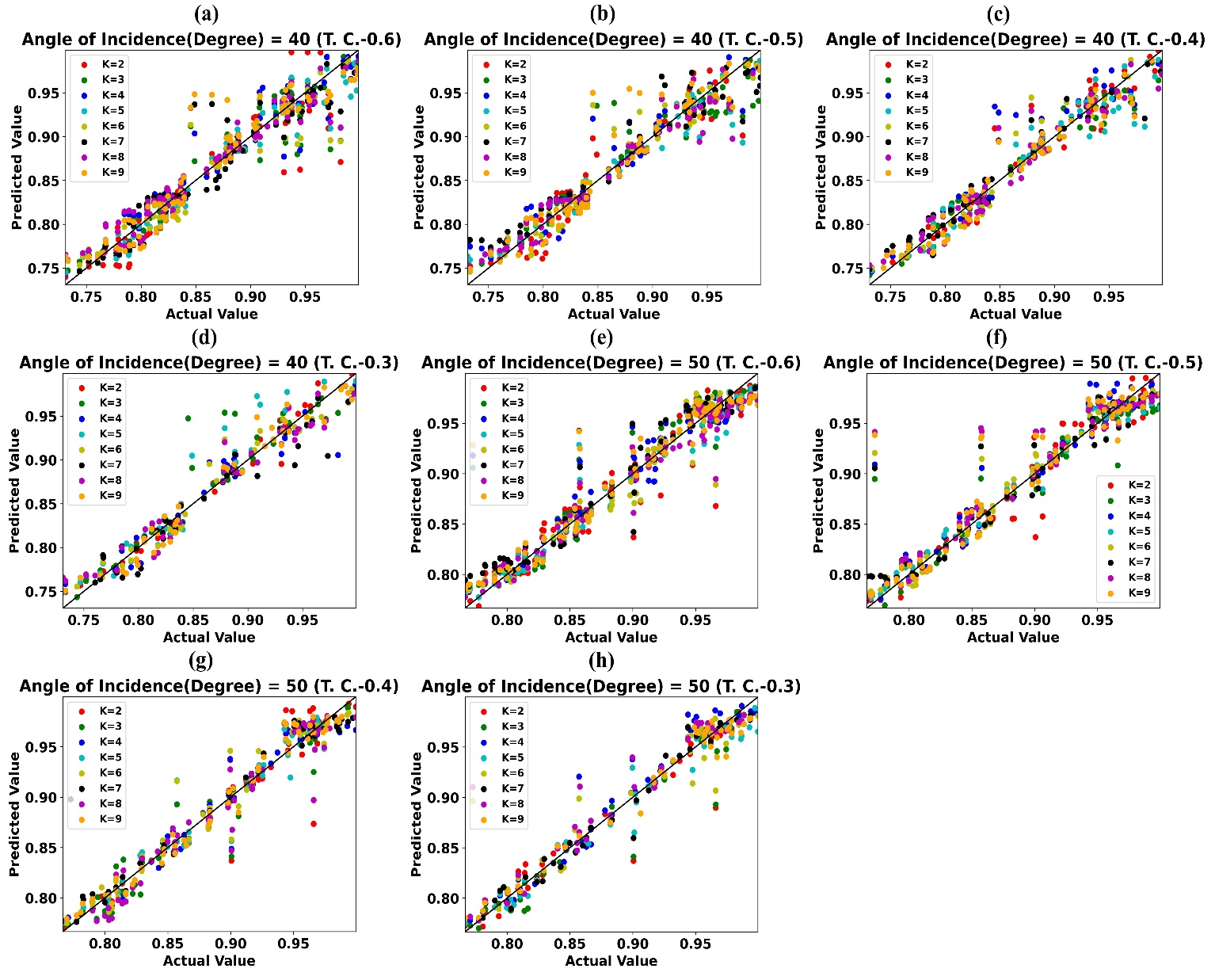


**Fig. S6** Predicted absorption value by weighted KNN-ressor vs actual absorption value for Angle of Incidence (degree) (a) 40^0^ (TC-0.6) (b) 40^0^ (TC-0.5) (c) 40^0^ (TC-0.4) (d) 40^0^ (TC-0.3) (e) 50^0^ (TC-0.6) (f) 50^0^ (TC-0.5) (g) 50^0^ (TC-0.4) (h) 50^0^ (TC-0.3)


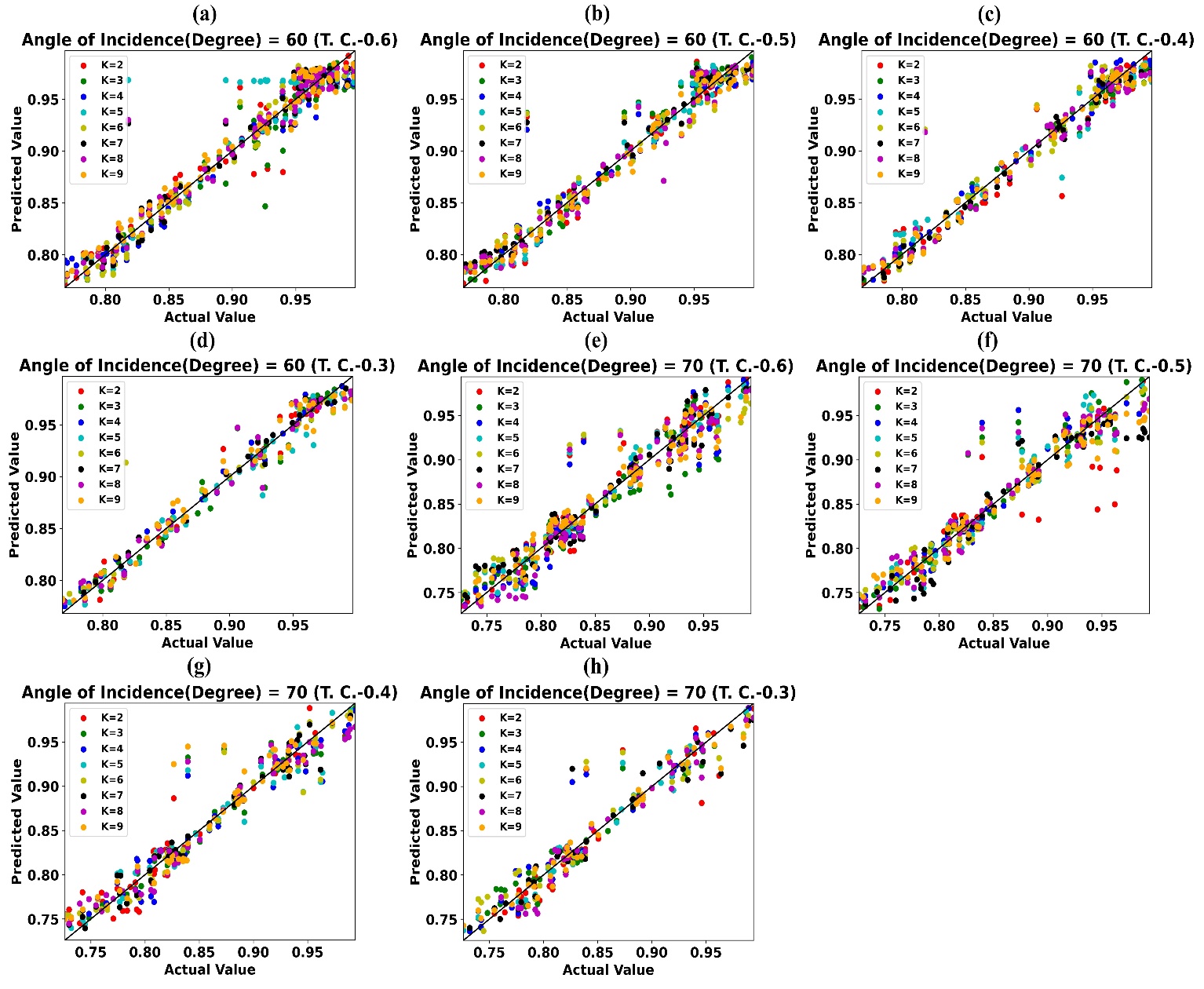


**Fig. S7** Predicted absorption value by weighted KNN-ressor vs actual absorption value for Angle of Incidence (degree) (a) 60^0^ (TC-0.6) (b) 60^0^ (TC-0.5) (c) 60^0^ (TC-0.4) (d) 60^0^ (TC-0.3) (e) 70^0^ (TC-0.6) (f) 70^0^ (TC-0.5) (g) 70^0^ (TC-0.4) (h) 70^0^ (TC-0.3)


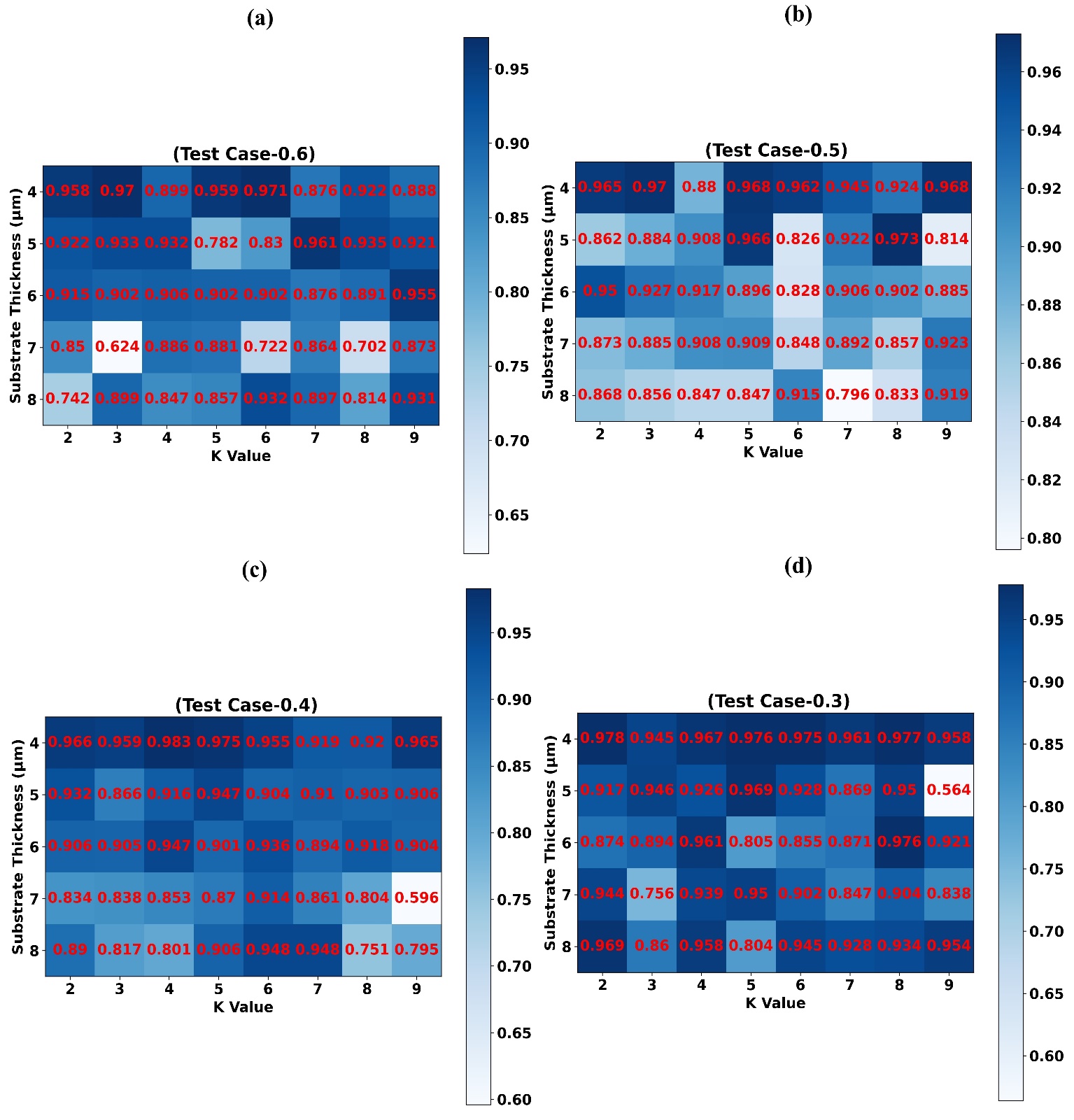


**Fig. S8** Prediction effectiveness (R2 Score) of Weighted KNN-regressor models trained for various values of Substrate Thickness (µm) and (a) TC-0.6 (b) TC-0.5 (c) TC-0.4 (d) TC-0.3


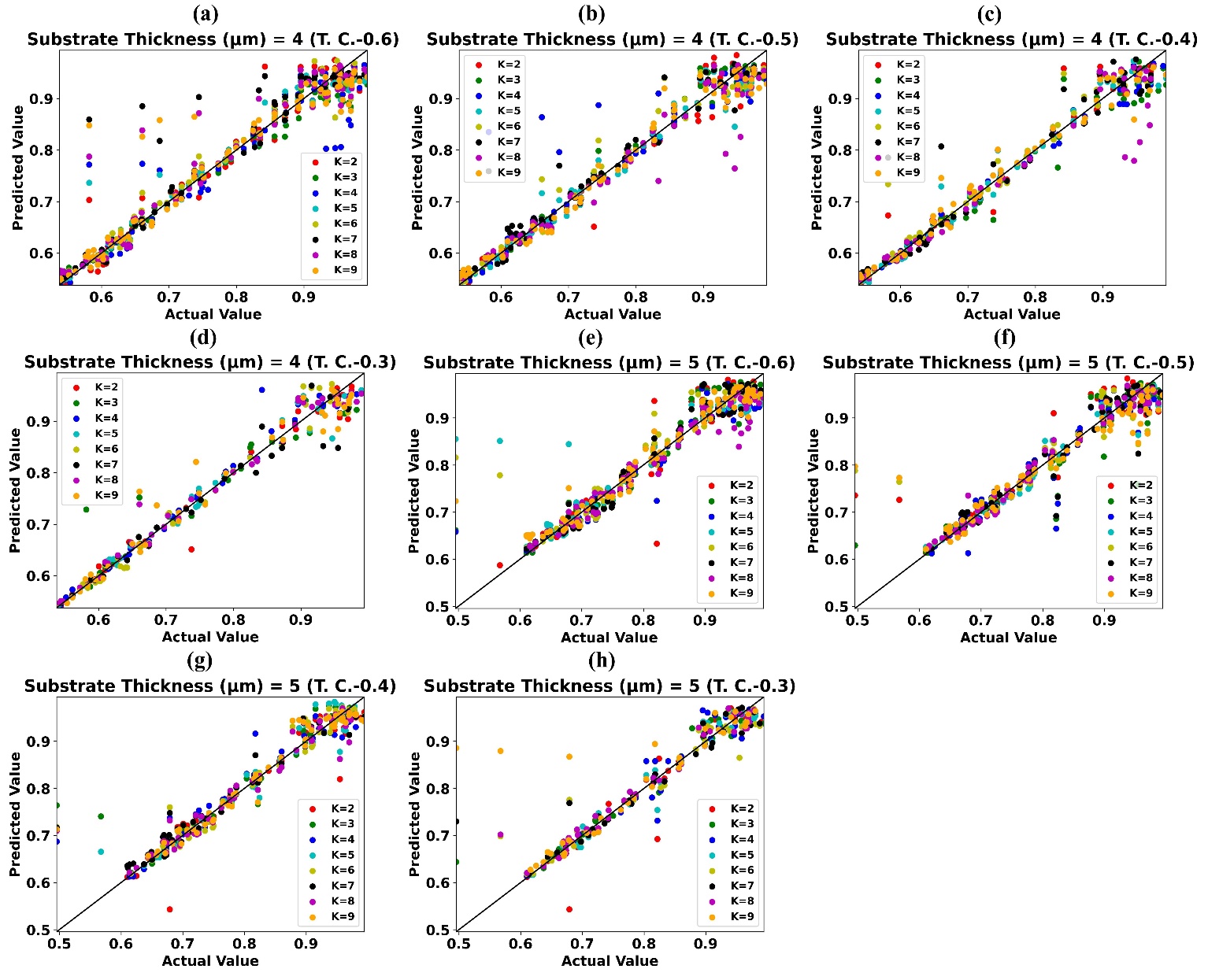


**Fig. S9** Predicted absorption value by weighted KNN-regressor vs actual absorption value for Substrate Thickness (µm) (a) 4 (TC-0.6) (b) 4 (TC-0.5) (c) 4 (TC-0.4) (d) 4 (TC-0.3) (e) 5 (TC-0.6) (f) 5 (TC-0.5) (g) 5 (TC-0.4) (h) 5 (TC-0.3)


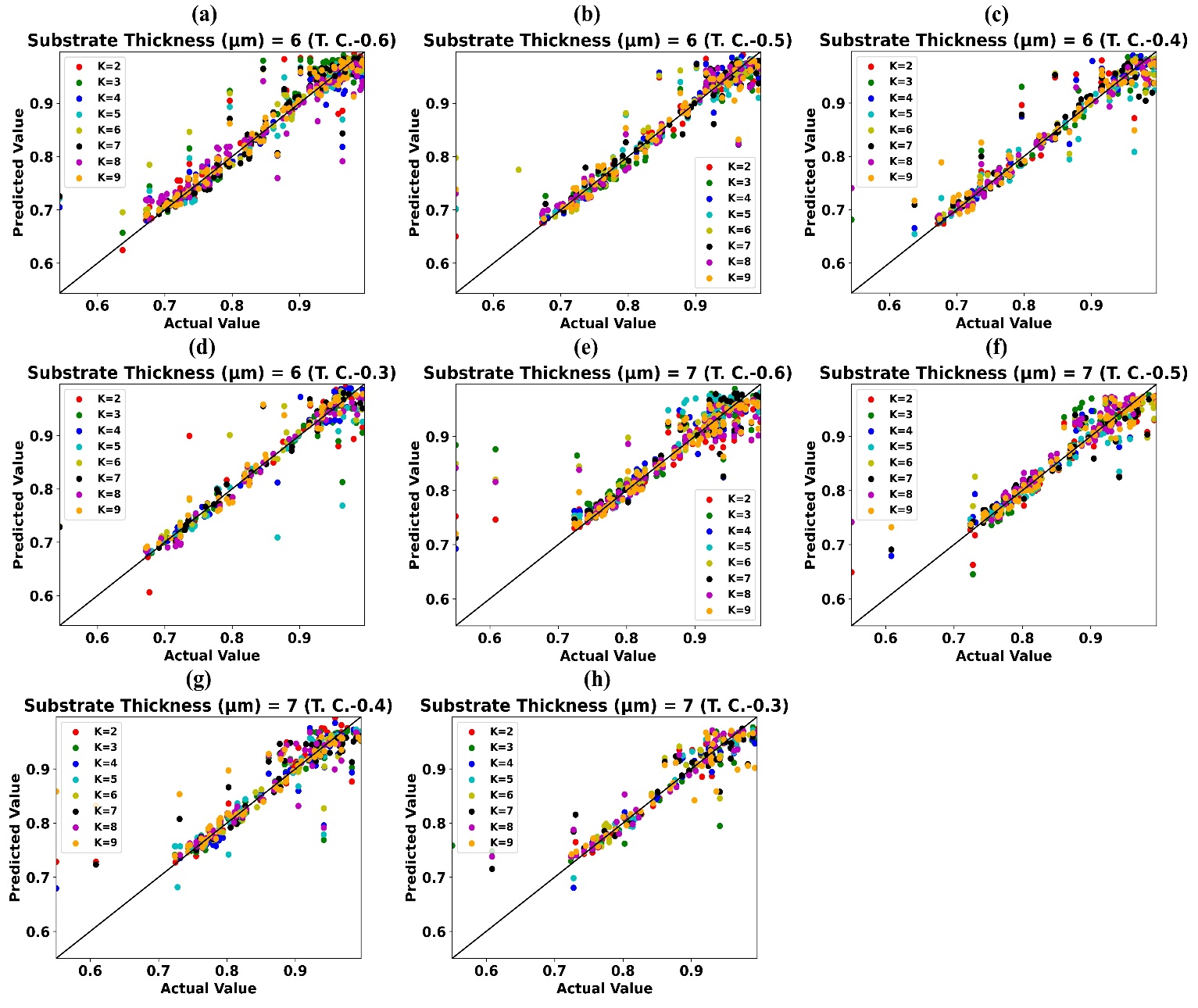


**Fig. S10** Predicted absorption value by weighted KNN-ressor vs actual absorption value for Substrate Thickness (µm) (a) 6 (TC-0.6) (b) 6 (TC-0.5) (c) 6 (TC-0.4) (d) 6 (TC-0.3) (e) 7 (TC-0.6) (f) 7 (TC-0.5) (g) 7 (TC-0.4) (h) 7 (TC-0.3)


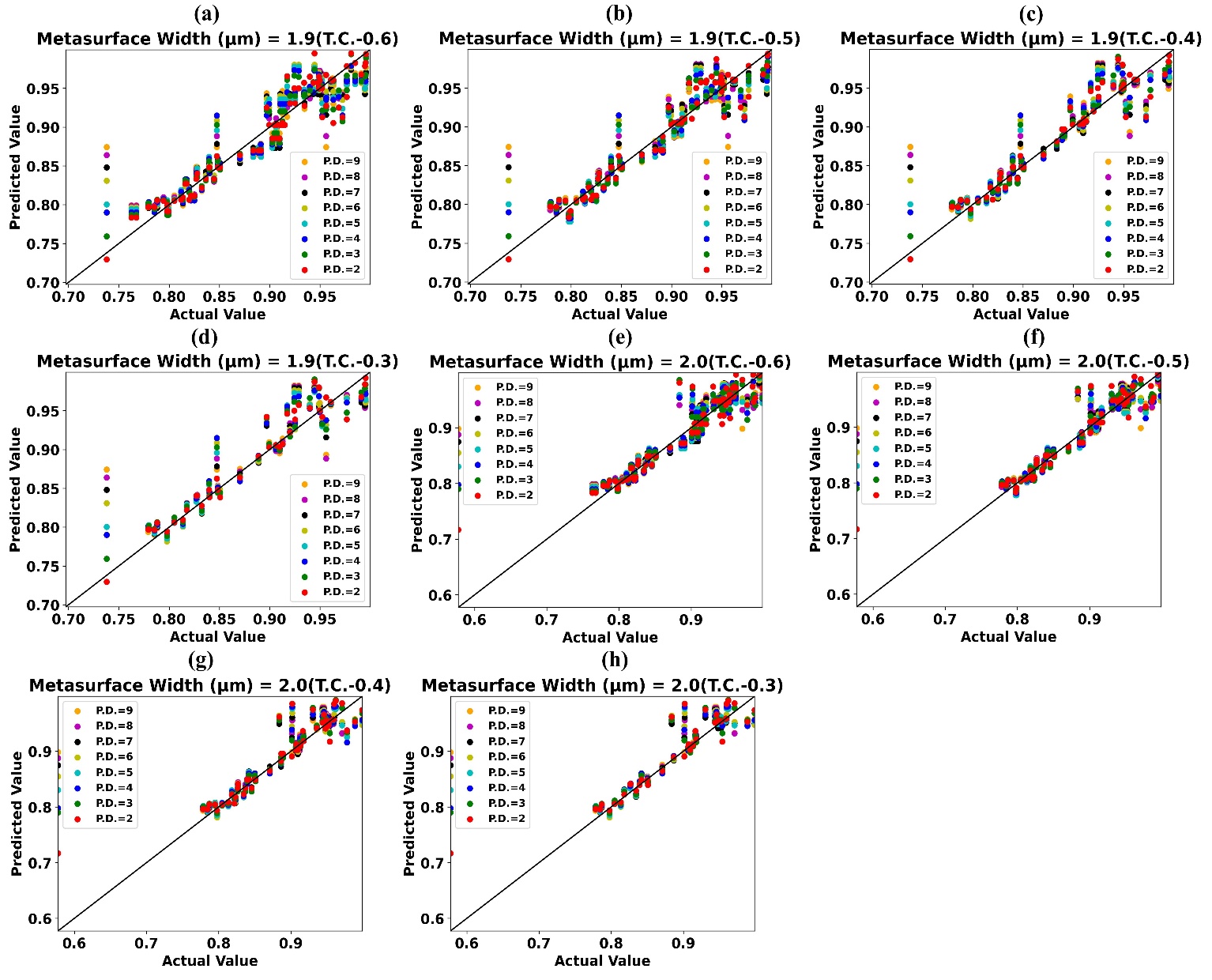


**Fig. S11** Predicted values of absorption by trained weighted KNN-regressor vs simulated/actual values of absorption for Metasurface Width (µm) (a) 1.9 (TC-0.6) (b) 1.9 (TC-0.5) (c) 1.9 (TC-0.4) (d) 1.9 (TC-0.3) (e) 2.0 (TC-0.6) (f) 2.0 (TC-0.5) (g) 2.0 (TC-0.4) (h) 2.0 (TC-0.3)


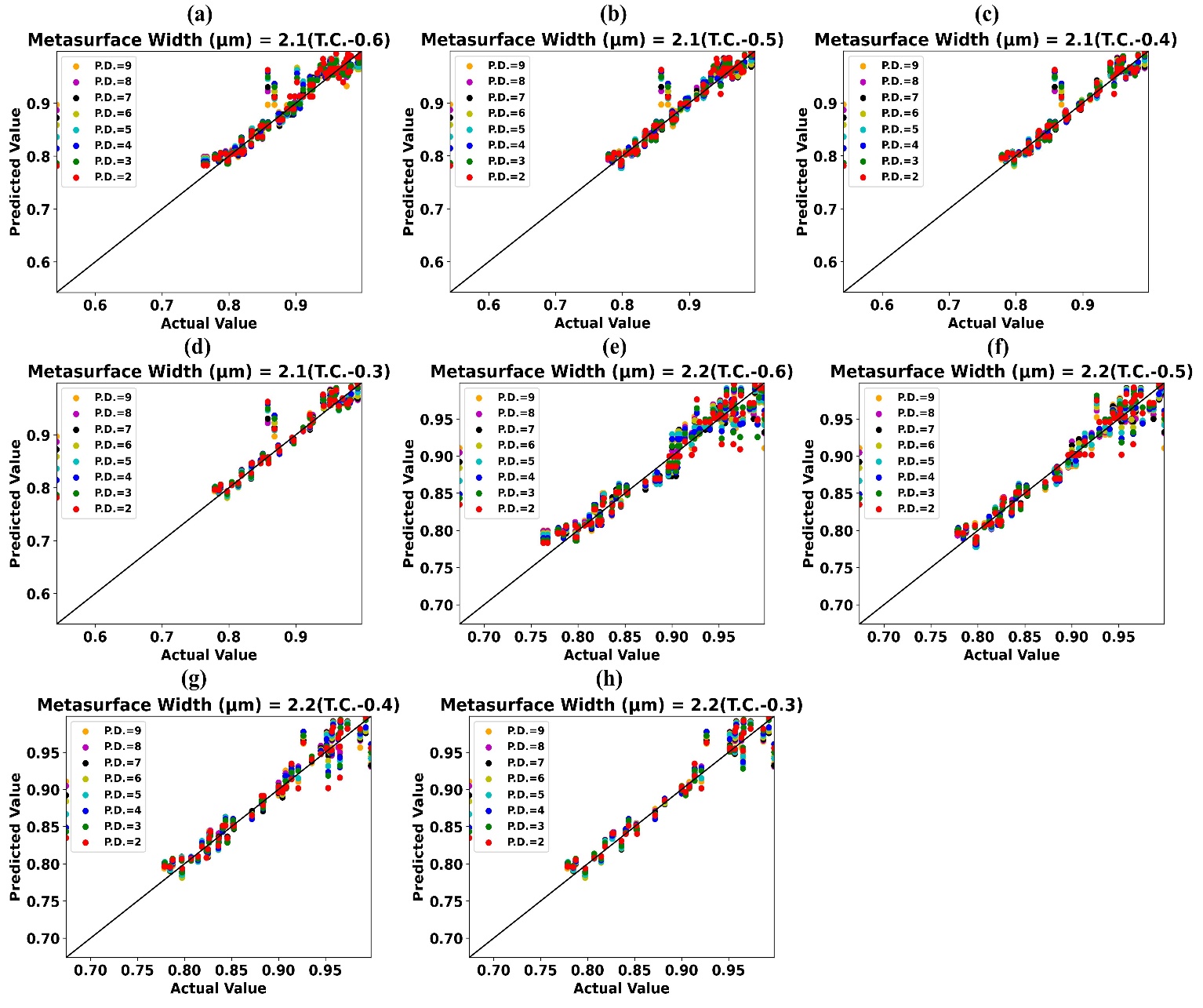


**Fig. S12** Predicted values of absorption by trained weighted KNN-regressor vs simulated/actual values of absorption for Metasurface Width (µm) (a) 2.1 (TC-0.6) (b) 2.1 (TC-0.5) (c) 2.1 (TC-0.4) (d) 2.1 (TC-0.3) (e) 2.2 (TC-0.6) (f) 2.2 (TC-0.5) (g) 2.2 (TC-0.4) (h) 2.2 (TC-0.3)


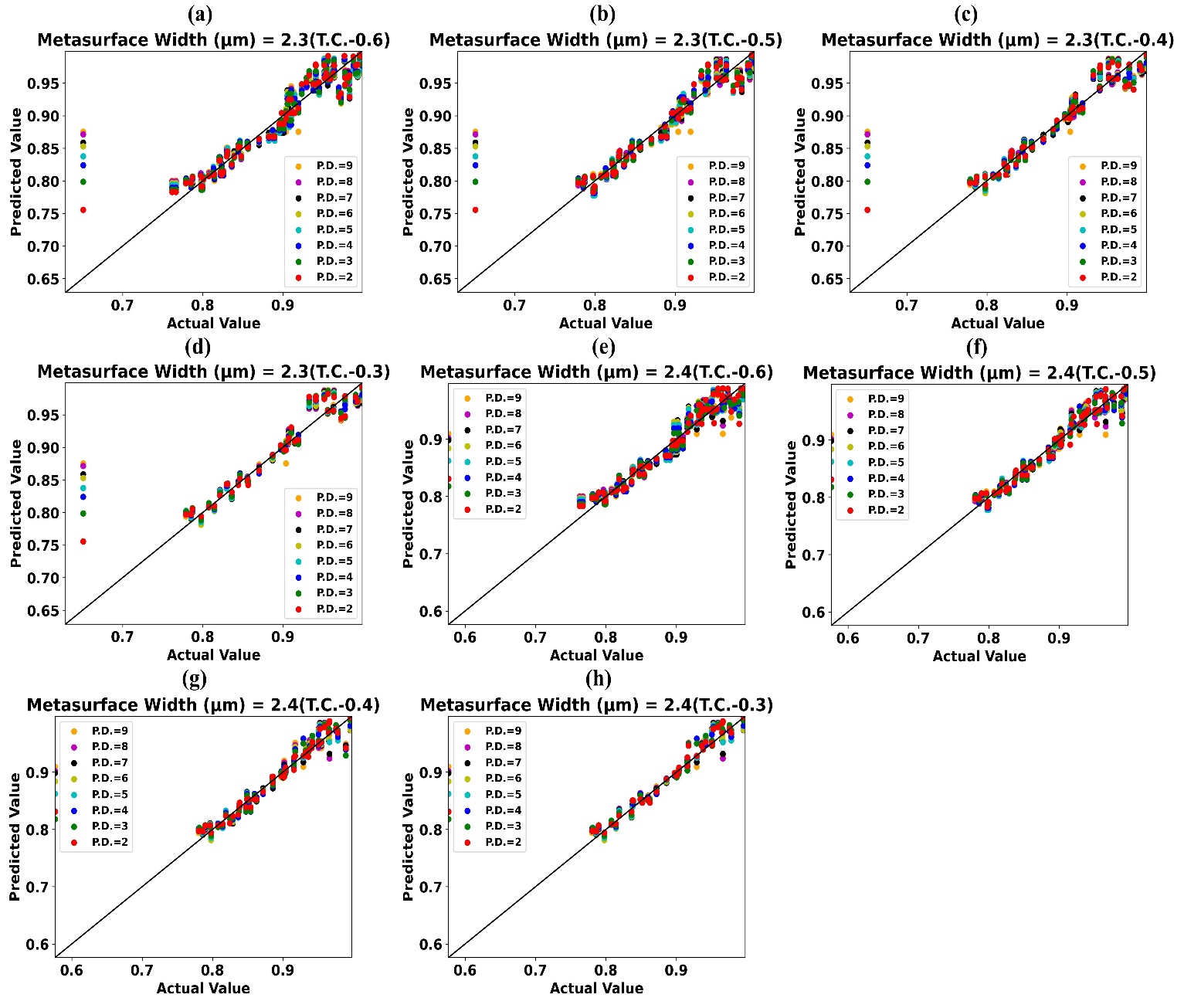


**Fig. S13** Predicted values of absorption by trained weighted KNN-regressor vs simulated/actual values of absorption for Metasurface Width (µm) (a) 2.3 (TC-0.6) (b) 2.3 (TC-0.5) (c) 2.3 (TC-0.4) (d) 2.3 (TC-0.3) (e) 2.4 (TC-0.6) (f) 2.4 (TC-0.5) (g) 2.4 (TC-0.4) (h) 2.4 (TC-0.3)


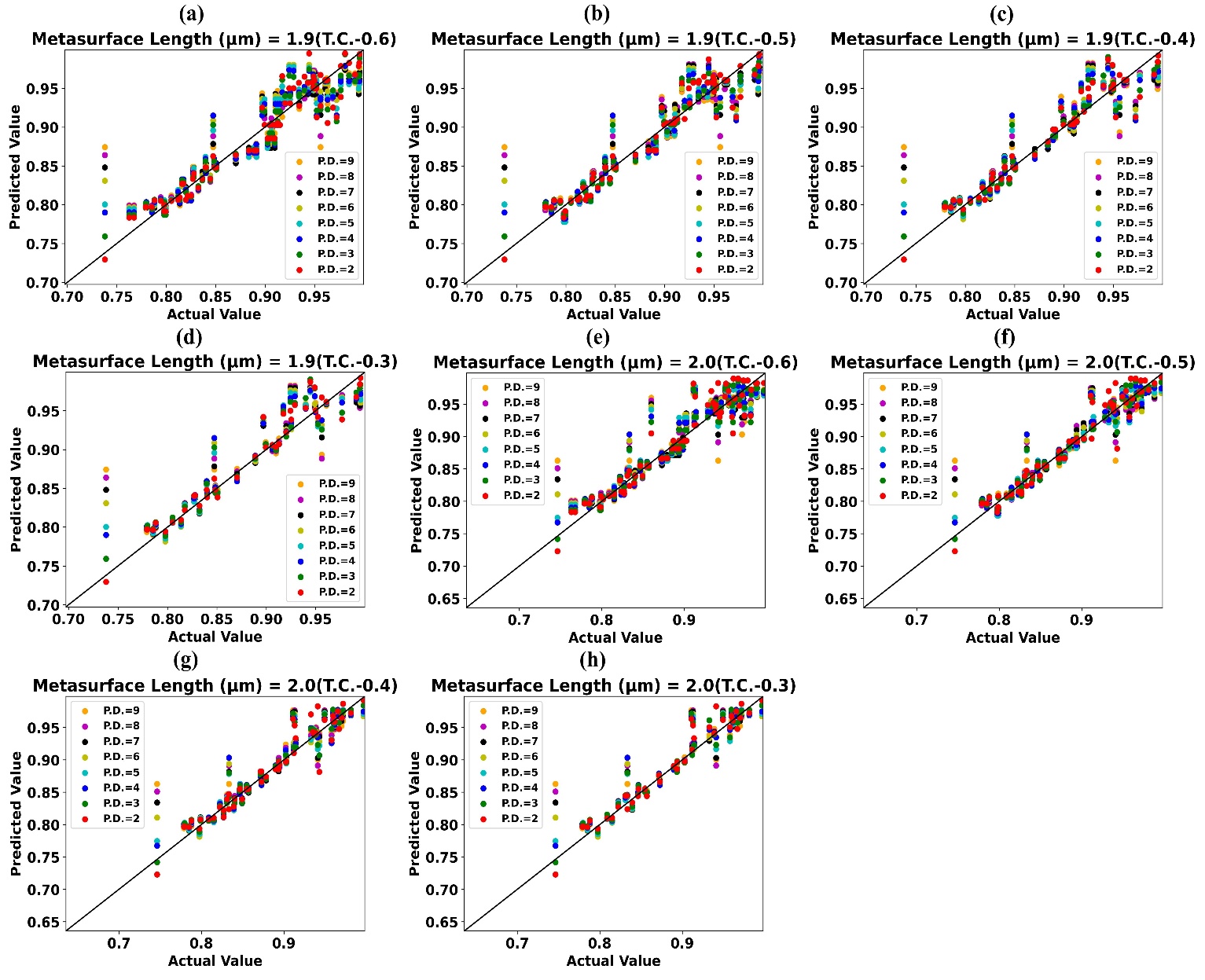


**Fig. S14** Predicted values of absorption by trained weighted KNN-regressor vs simulated/actual values of absorption for Metasurface Length (µm) (a) 1.9 (TC-0.6) (b) 1.9 (TC-0.5) (c) 1.9 (TC-0.4) (d) 1.9 (TC-0.3) (e) 2.0 (TC-0.6) (f) 2.0 (TC-0.5) (g) 2.0 (TC-0.4) (h) 2.0 (TC-0.3)


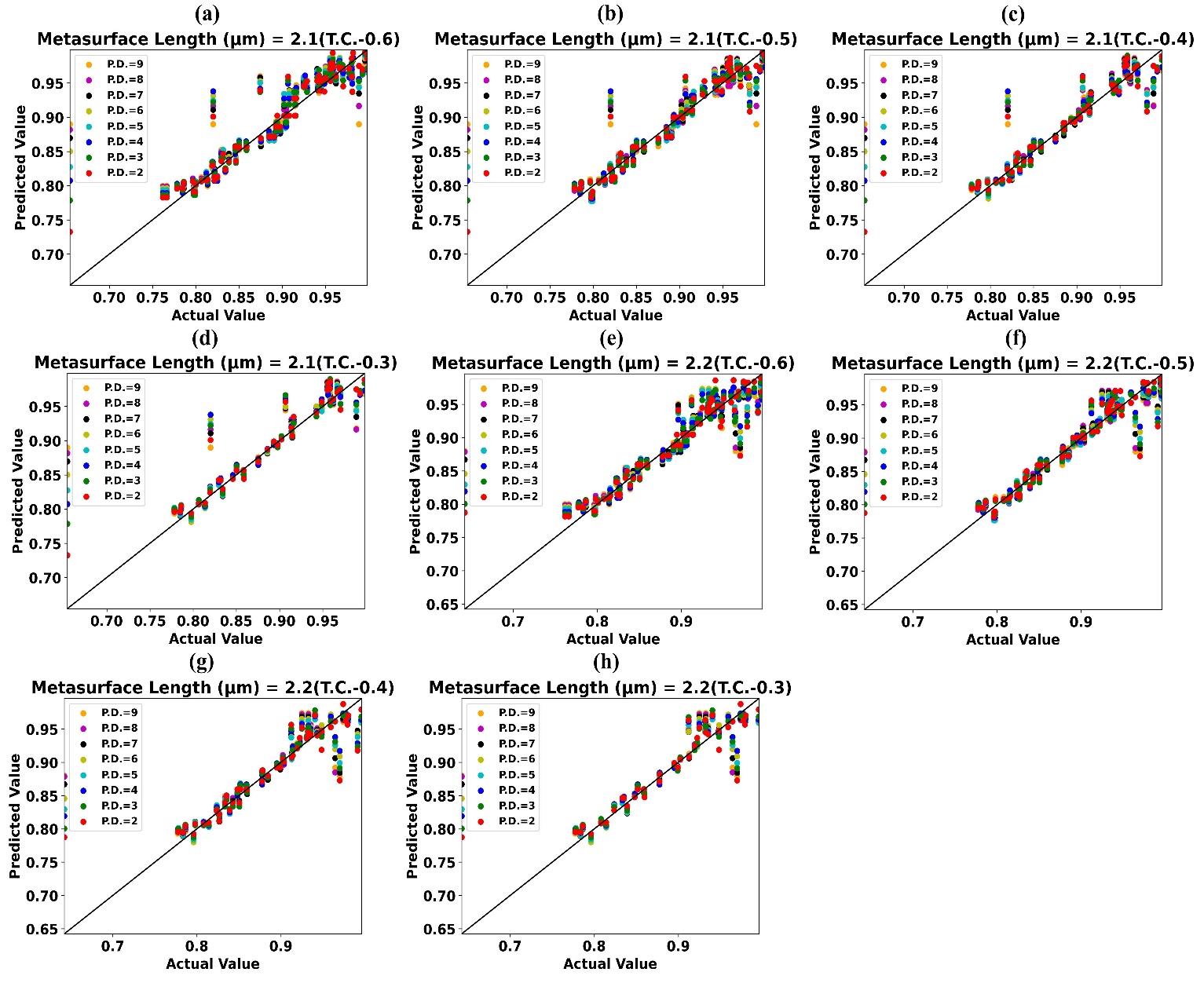


**Fig. S15** Predicted values of absorption by trained weighted KNN-regressor vs simulated/actual values of absorption for Metasurface Length (µm) (a) 2.1 (TC-0.6) (b) 2.1 (TC-0.5) (c) 2.1 (TC-0.4) (d) 2.1 (TC-0.3) (e) 2.2 (TC-0.6) (f) 2.2 (TC-0.5) (g) 2.2 (TC-0.4) (h) 2.2 (TC-0.3)


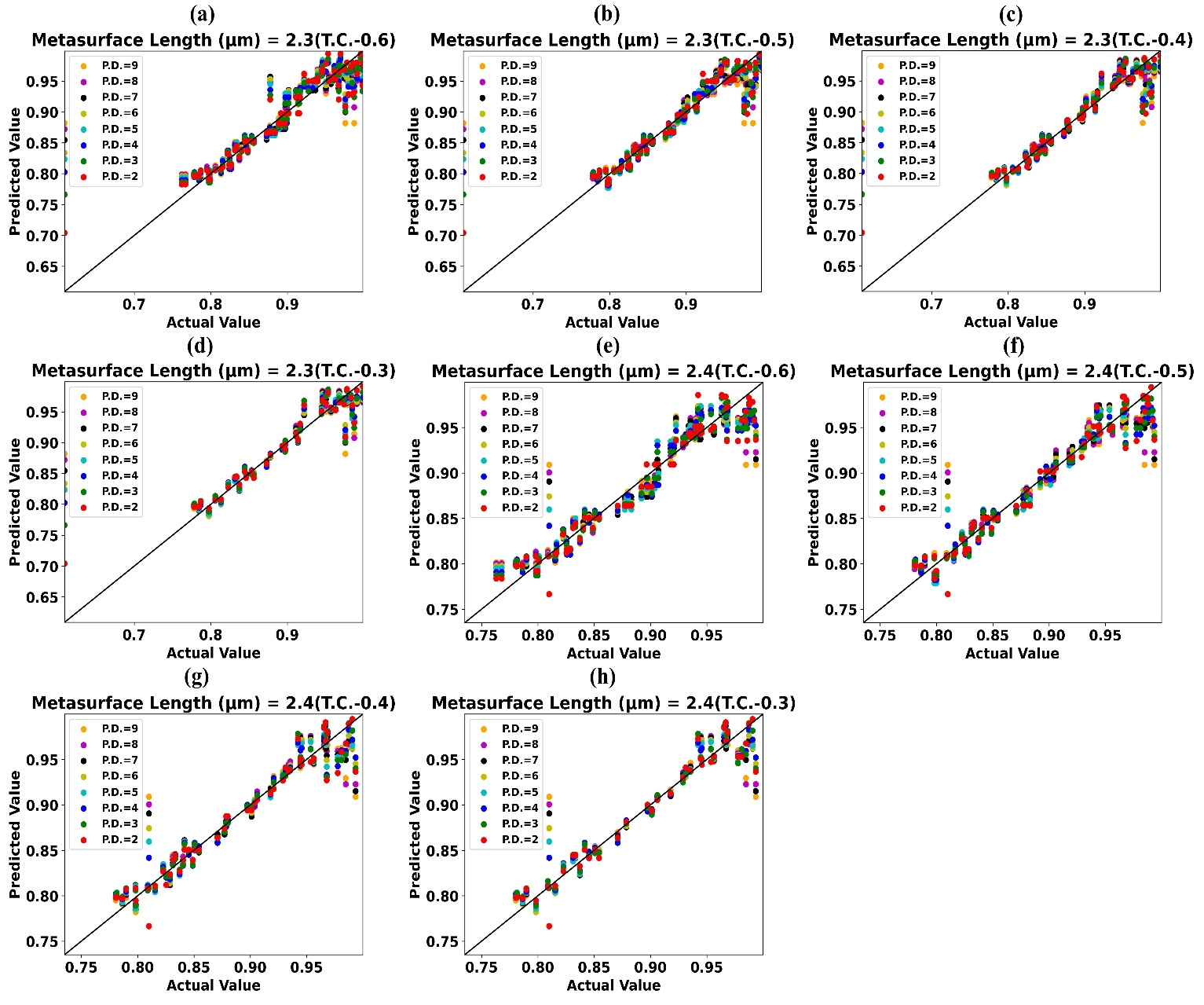


**Fig. S16** Predicted values of absorption by trained weighted KNN-regressor vs simulated/actual values of absorption for Metasurface Length (µm) (a) 2.3 (TC-0.6) (b) 2.3 (TC-0.5) (c) 2.3 (TC-0.4) (d) 2.3 (TC-0.3) (e) 2.4 (TC-0.6) (f) 2.4 (TC-0.5) (g) 2.4 (TC-0.4) (h) 2.4 (TC-0.3)


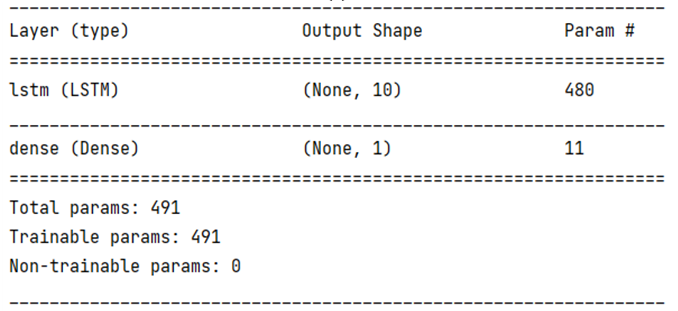


**Fig. 17** Layer Structure of LSTM Model used for forecasting Values of absorption for forthcoming wavelengths


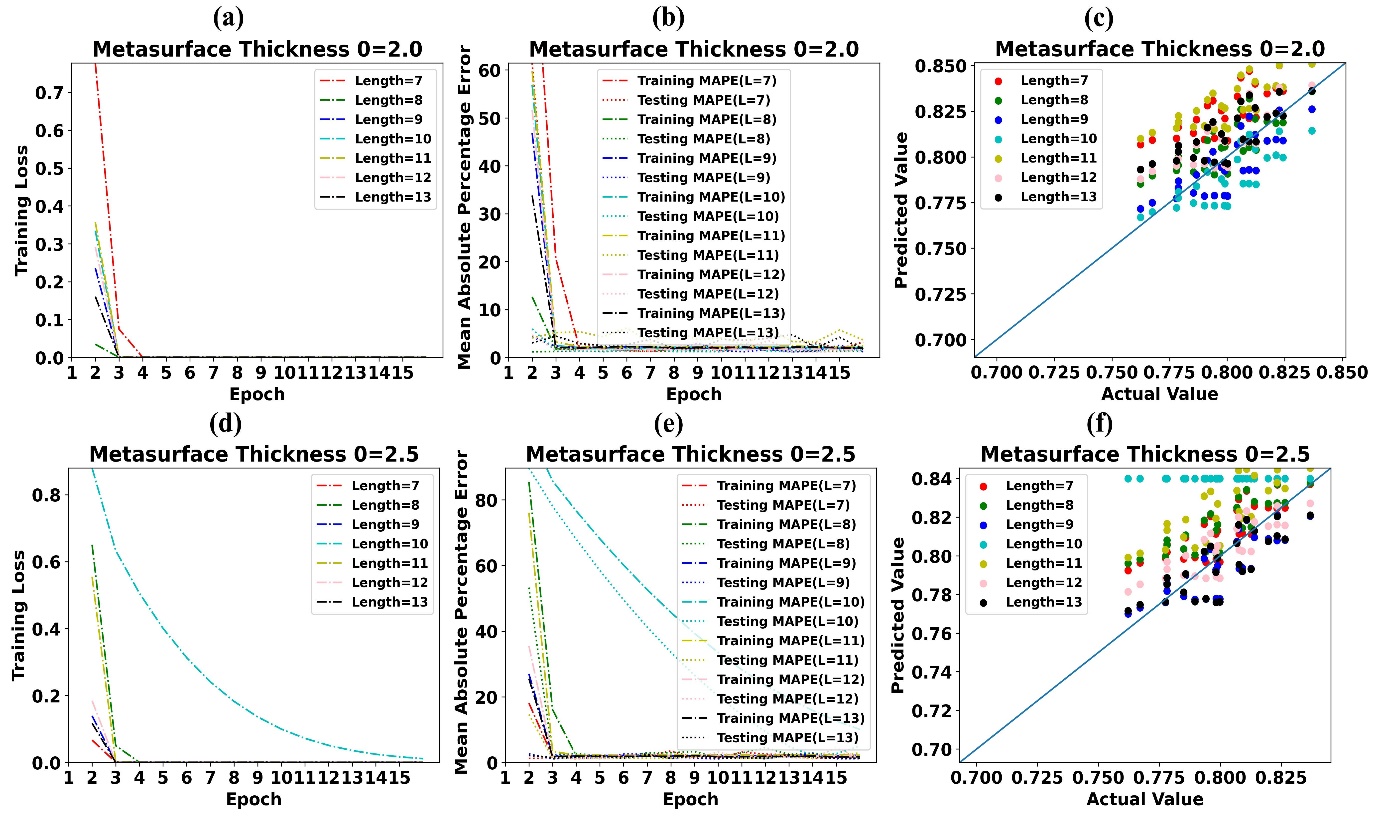


**Fig. S18** (a) Training Loss of LSTM model for Metasurface Thickness 2.0 µm (b) MAPE of LSTM model for Metasurface Thickness 2.0 µm (c) Predicted values of absorption by LSTM vs simulated values of absorption for Metasurface Thickness 2.0 µm (d) Training Loss of LSTM model for Metasurface Thickness 2.5 µm (e) MAPE of LSTM model for Metasurface Thickness 2.5 µm (f) Predicted values of absorption by LSTM vs simulated values of absorption for Metasurface Thickness 2.5 µm


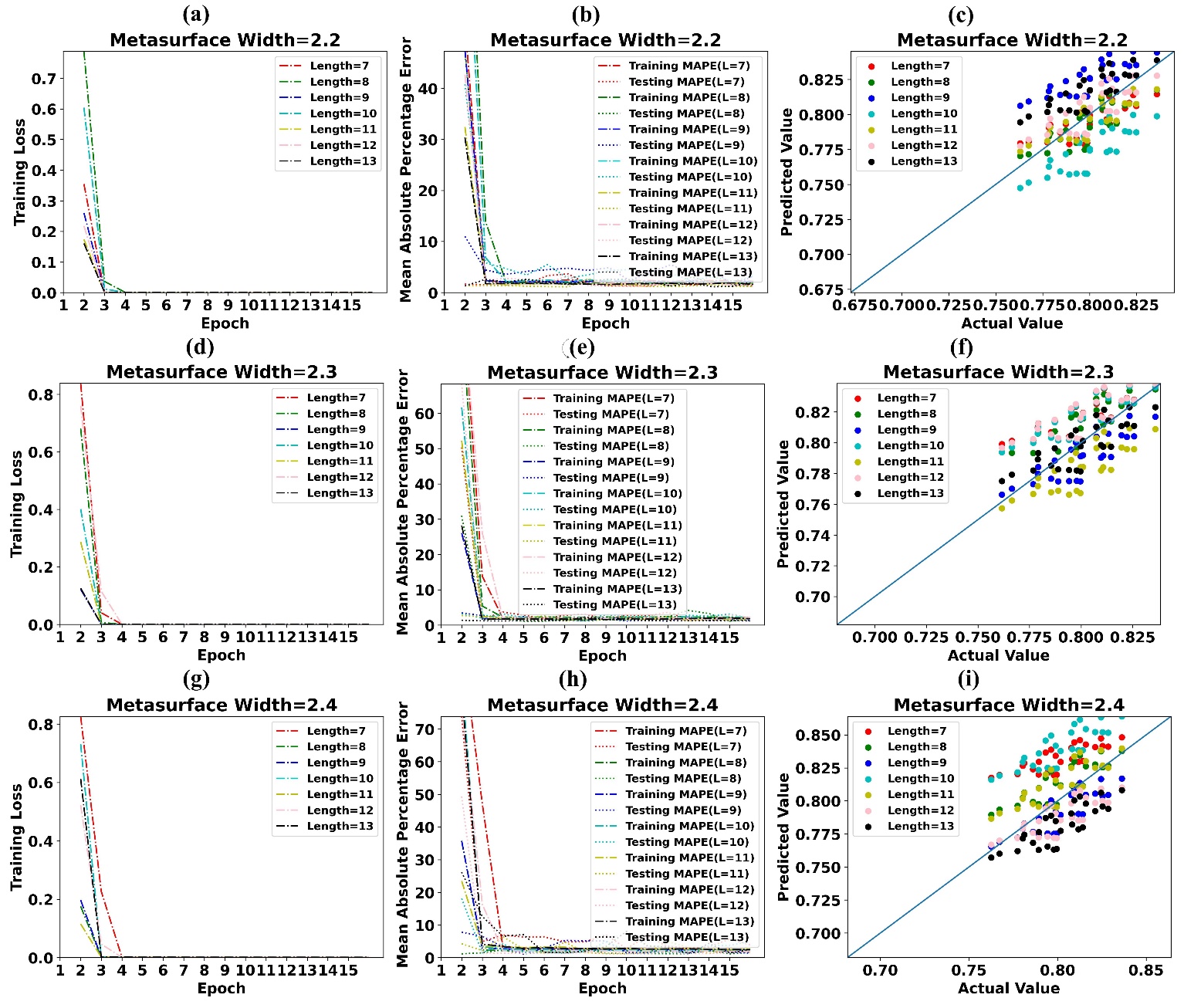


**Fig. S19** (a) Training Loss of LSTM model for Metasurface Width 2.2 µm (b) MAPE of LSTM model for Metasurface Width 2.2 µm (c) Predicted values of absorption by LSTM vs simulated values of absorption for Metasurface Width 2.2 µm (d) Training Loss of LSTM model for Metasurface Width 2.3 µm (e) MAPE of LSTM model for Metasurface Width 2.3 µm (f) Predicted values of absorption by LSTM vs simulated values of absorption for Metasurface Width 2.3 µm (g) Training Loss of LSTM model for Metasurface Width 2.4 µm (h) MAPE of LSTM model for Metasurface Width 2.4 µm (i) Predicted values of absorption by LSTM vs simulated values of absorption for Metasurface Width 2.4 µm


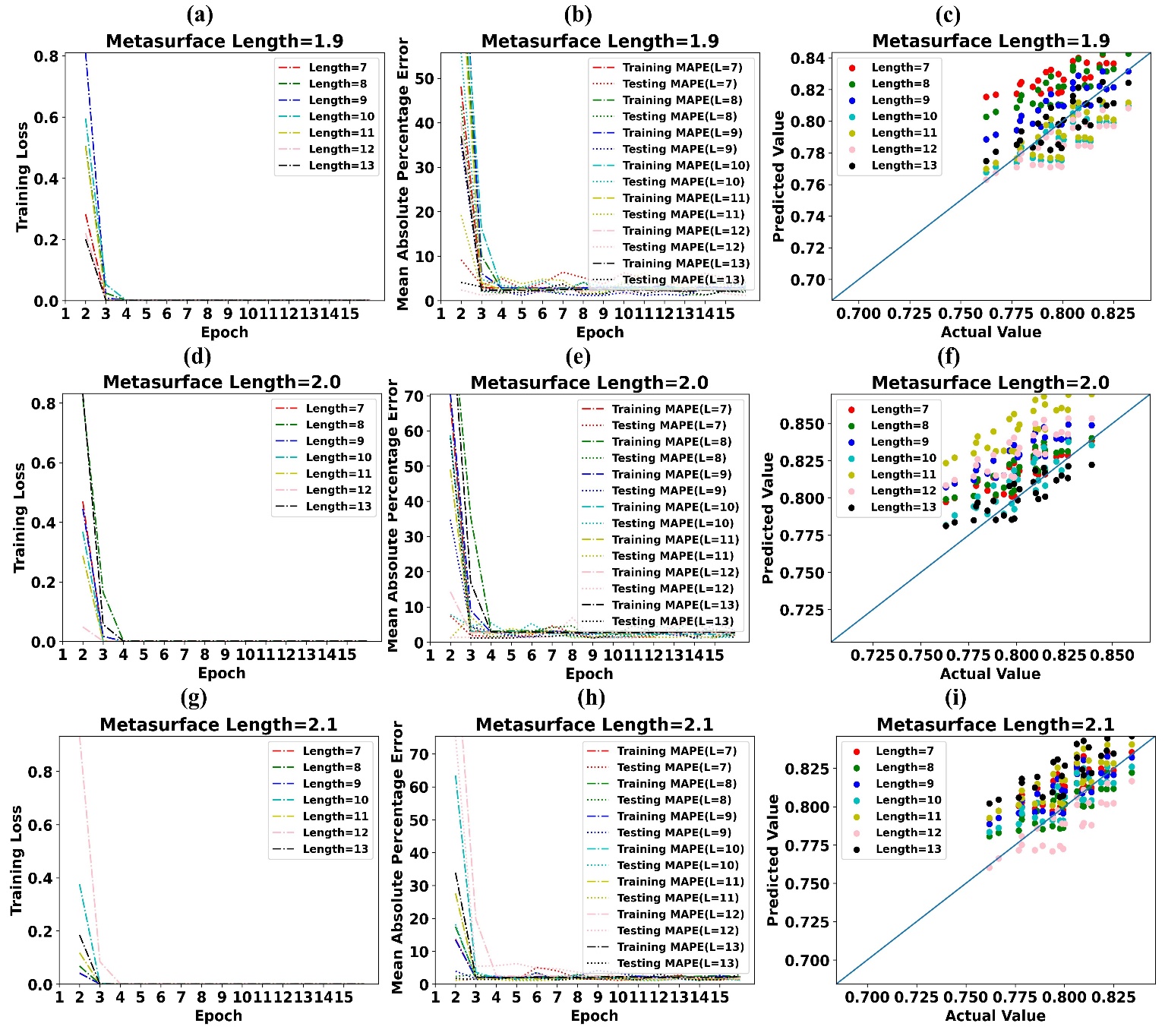


**Fig. S20** (a) Training Loss of LSTM model for Metasurface Length 1.9 µm (b) MAPE of LSTM model for Metasurface Length 1.9 µm (c) Predicted values of absorption by LSTM vs simulated values of absorption for Metasurface Length 1.9 µm (d) Training Loss of LSTM model for Metasurface Length 2.0 µm (e) MAPE of LSTM model for Metasurface Length 2.0 µm (f) Predicted values of absorption by LSTM vs simulated values of absorption for Metasurface Length 2.0 µm (g) Training Loss of LSTM model for Metasurface Length 2.1 µm (h) MAPE of LSTM model for Metasurface Length 2.1 µm (i) Predicted values of absorption by LSTM vs simulated values of absorption for Metasurface Length 2.1 µm


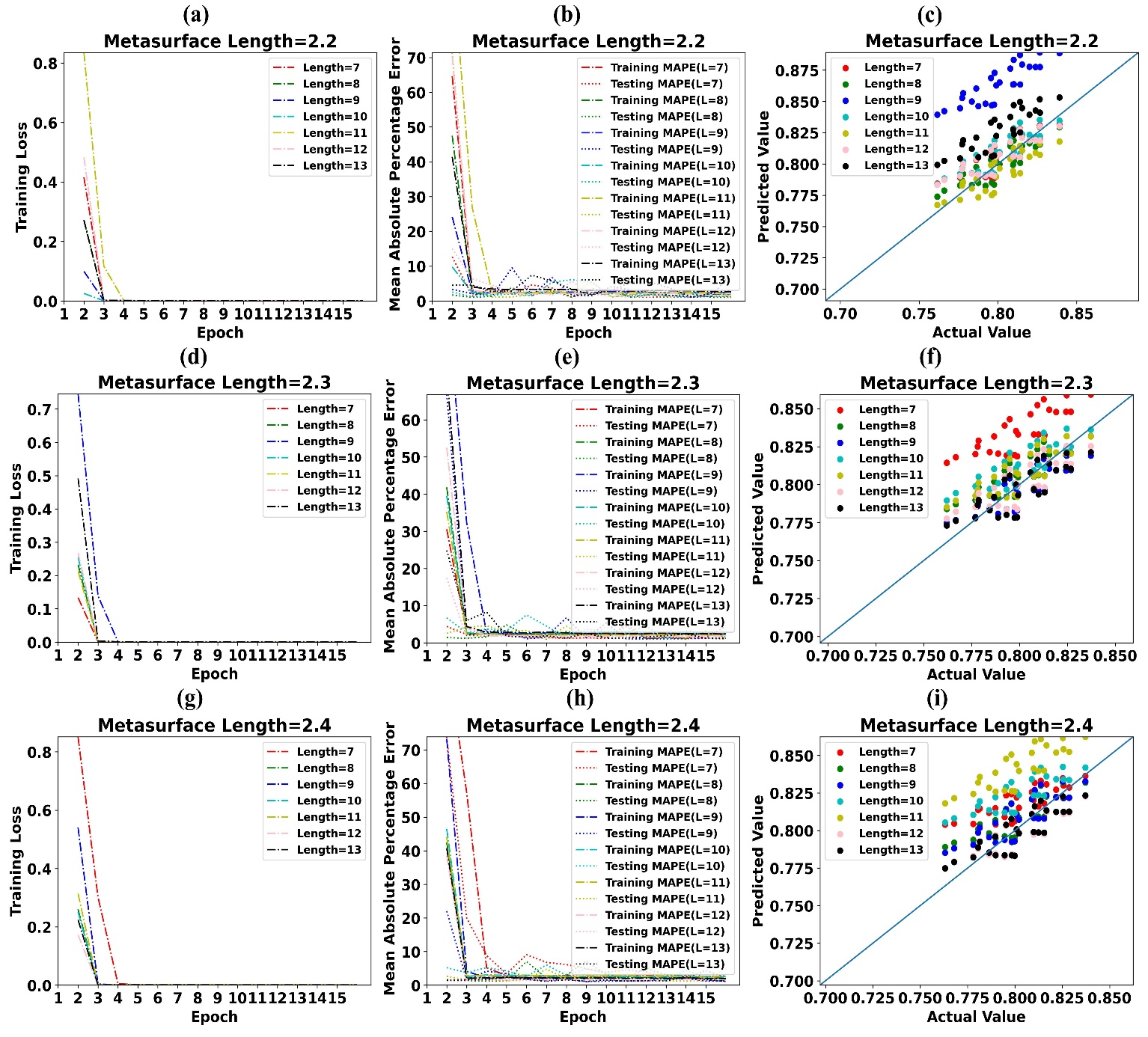


**Fig. S21** (a) Training Loss of LSTM model for Metasurface Length 2.2 µm (b) MAPE of LSTM model for Metasurface Length 2.2 µm (c) Predicted values of absorption by LSTM vs simulated values of absorption for Metasurface Length 2.2 µm (d) Training Loss of LSTM model for Metasurface Length 2.3 µm (e) MAPE of LSTM model for Metasurface Length 2.3 µm (f) Predicted values of absorption by LSTM vs simulated values of absorption for Metasurface Length 2.3 µm (g) Training Loss of LSTM model for Metasurface Length 2.4 µm (h) MAPE of LSTM model for Metasurface Length 2.4 µm (i) Predicted values of absorption by LSTM vs simulated values of absorption for Metasurface Length 2.4 µm


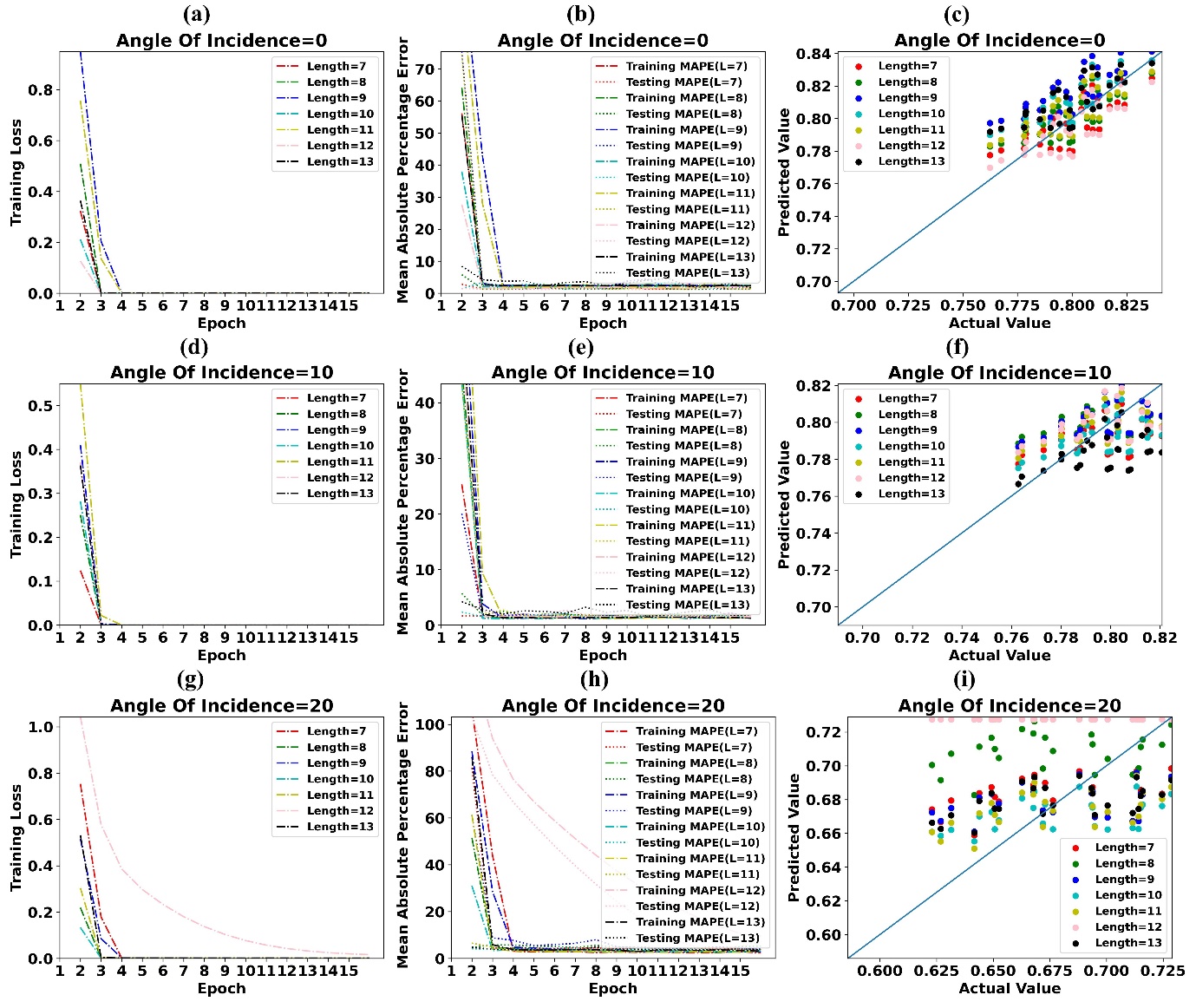


**Fig. S22** (a) Training Loss of LSTM model for Angle of Incidence 0^0^ (b) MAPE of LSTM model for Angle of Incidence 0^0^ (c) Predicted values of absorption by LSTM vs simulated values of absorption for Angle of Incidence 0^0^ (d) Training Loss of LSTM model for Angle of Incidence 10^0^ (e) MAPE of LSTM model for Angle of Incidence 10^0^ (f) Predicted values of absorption by LSTM vs simulated values of absorption for Angle of Incidence 10^0^ (g) Training Loss of LSTM model for Angle of Incidence 20^0^ (h) MAPE of LSTM model for Angle of Incidence 200 (i) Predicted values of absorption by LSTM vs simulated values of absorption for Angle of Incidence 20^0^


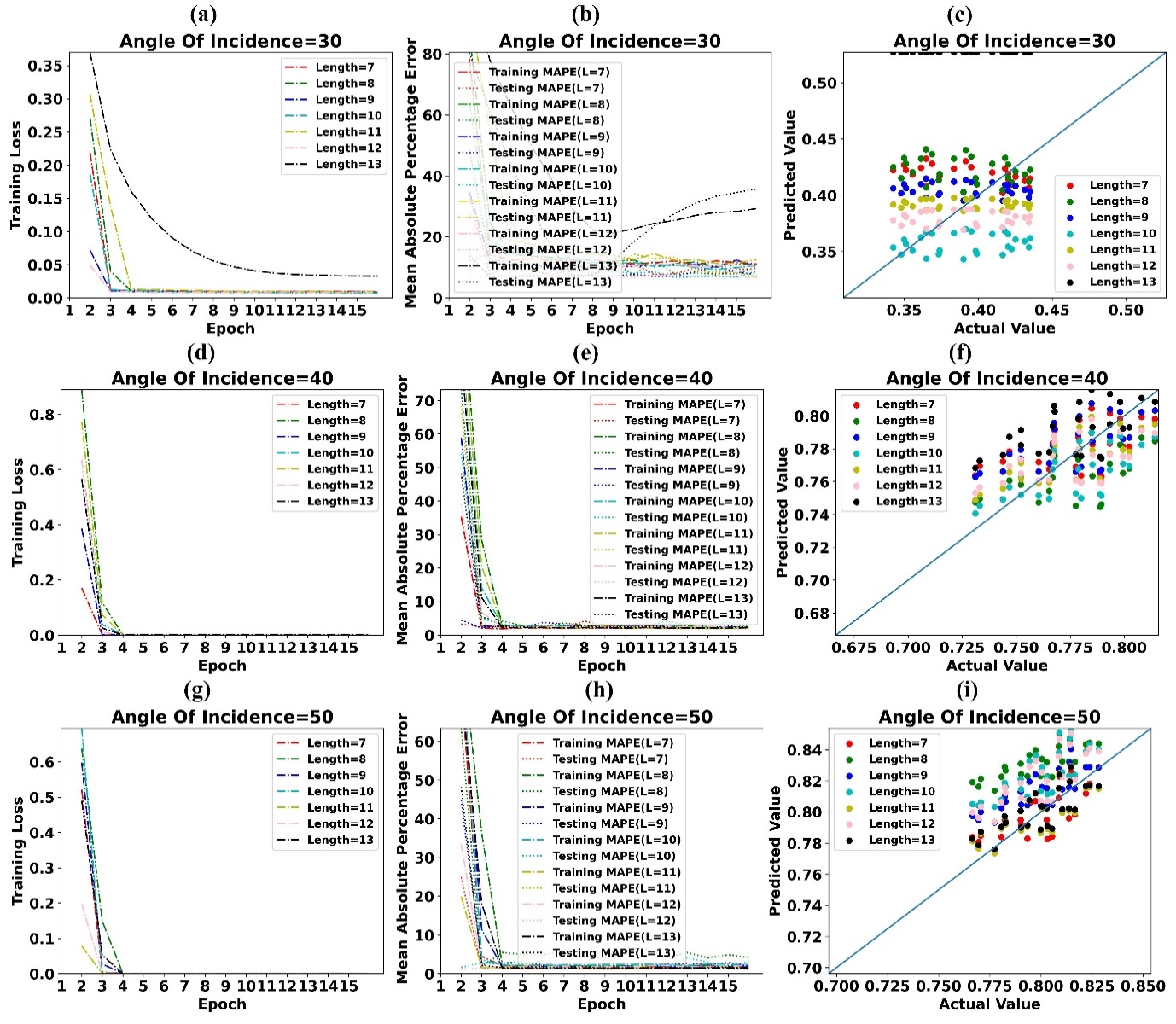


**Fig. S23** (a) Training Loss of LSTM model for Angle of Incidence 30^0^ (b) MAPE of LSTM model for Angle of Incidence 30^0^ (c) Predicted values of absorption by LSTM vs simulated values of absorption for Angle of Incidence 30^0^ (d) Training Loss of LSTM model for Angle of Incidence 40^0^ (e) MAPE of LSTM model for Angle of Incidence 40^0^ (f) Predicted values of absorption by LSTM vs simulated values of absorption for Angle of Incidence 40^0^ (g) Training Loss of LSTM model for Angle of Incidence 50^0^ (h) MAPE of LSTM model for Angle of Incidence 50^0^ (i) Predicted values of absorption by LSTM vs simulated values of absorption for Angle of Incidence 50^0^.


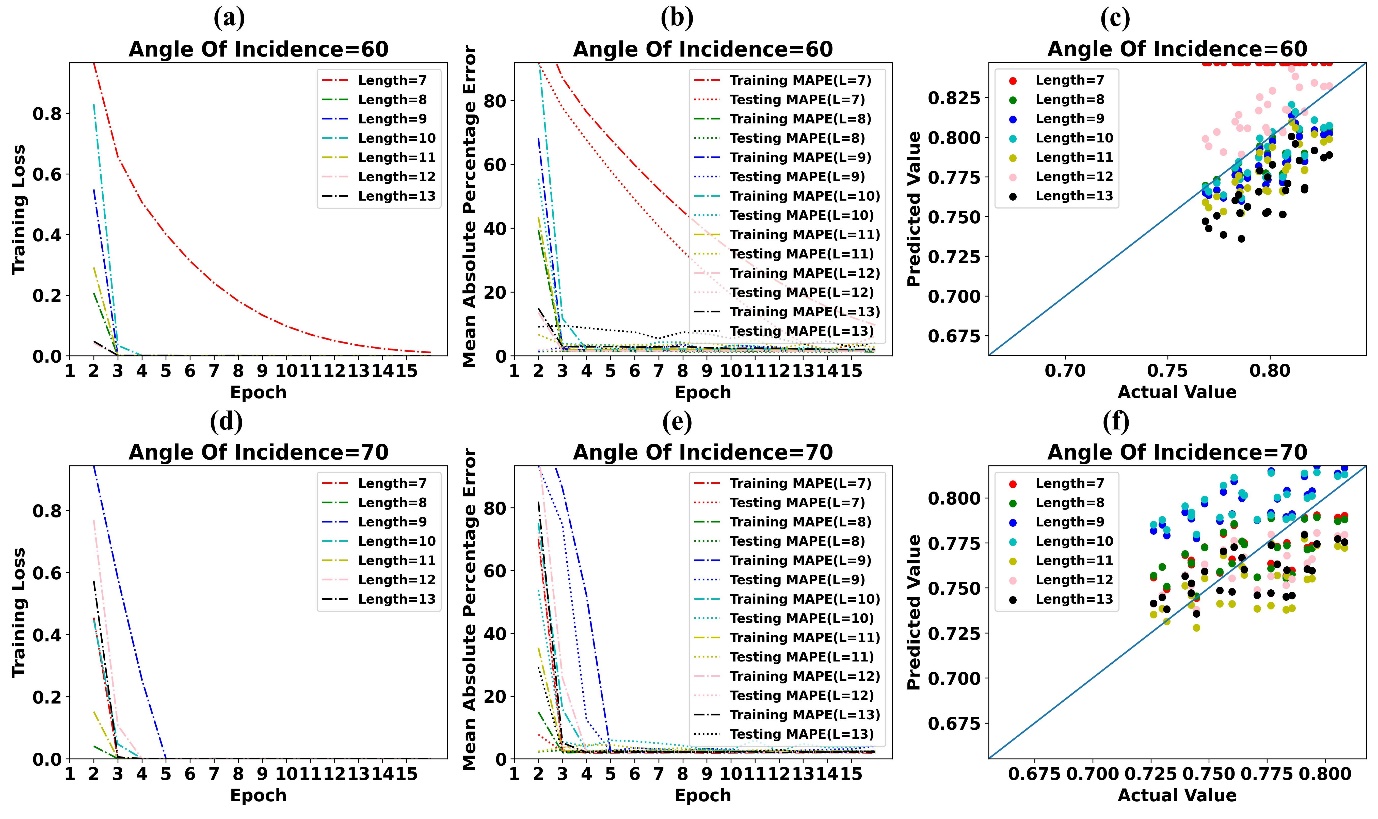


**Fig. S24** (a) Training Loss of LSTM model for Angle of Incidence 60^0^ (b) MAPE of LSTM model for Angle of Incidence 60^0^ (c) Predicted values of absorption by LSTM vs simulated values of absorption for Angle of Incidence 60^0^ (d) Training Loss of LSTM model for Angle of Incidence 70^0^ (e) MAPE of LSTM model for Angle of Incidence 70^0^ (f) Predicted values of absorption by LSTM vs simulated values of absorption for Angle of Incidence 70^0^


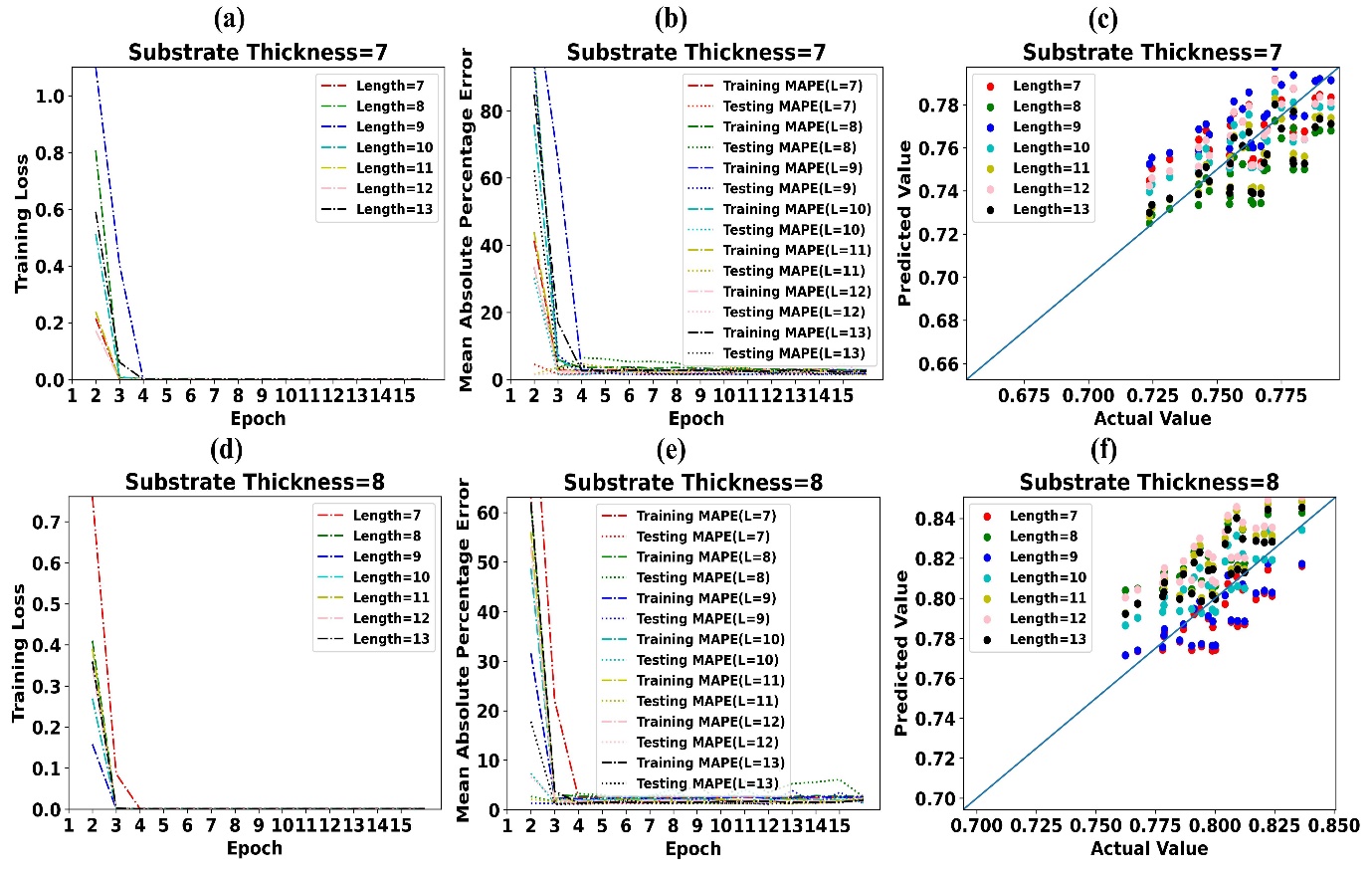


**Fig. S25** (a) Training Loss of LSTM model for Substrate Thickness 7 µm (b) MAPE of LSTM model for Substrate Thickness 7 µm (c) Predicted values of absorption by LSTM vs simulated values of absorption for Substrate Thickness 7 µm (d) Training Loss of LSTM model for Substrate Thickness 8 µm (e) MAPE of LSTM model for Substrate Thickness 8 µm (f) Predicted values of absorption by LSTM vs simulated values of absorption for Substrate Thickness 8 µm


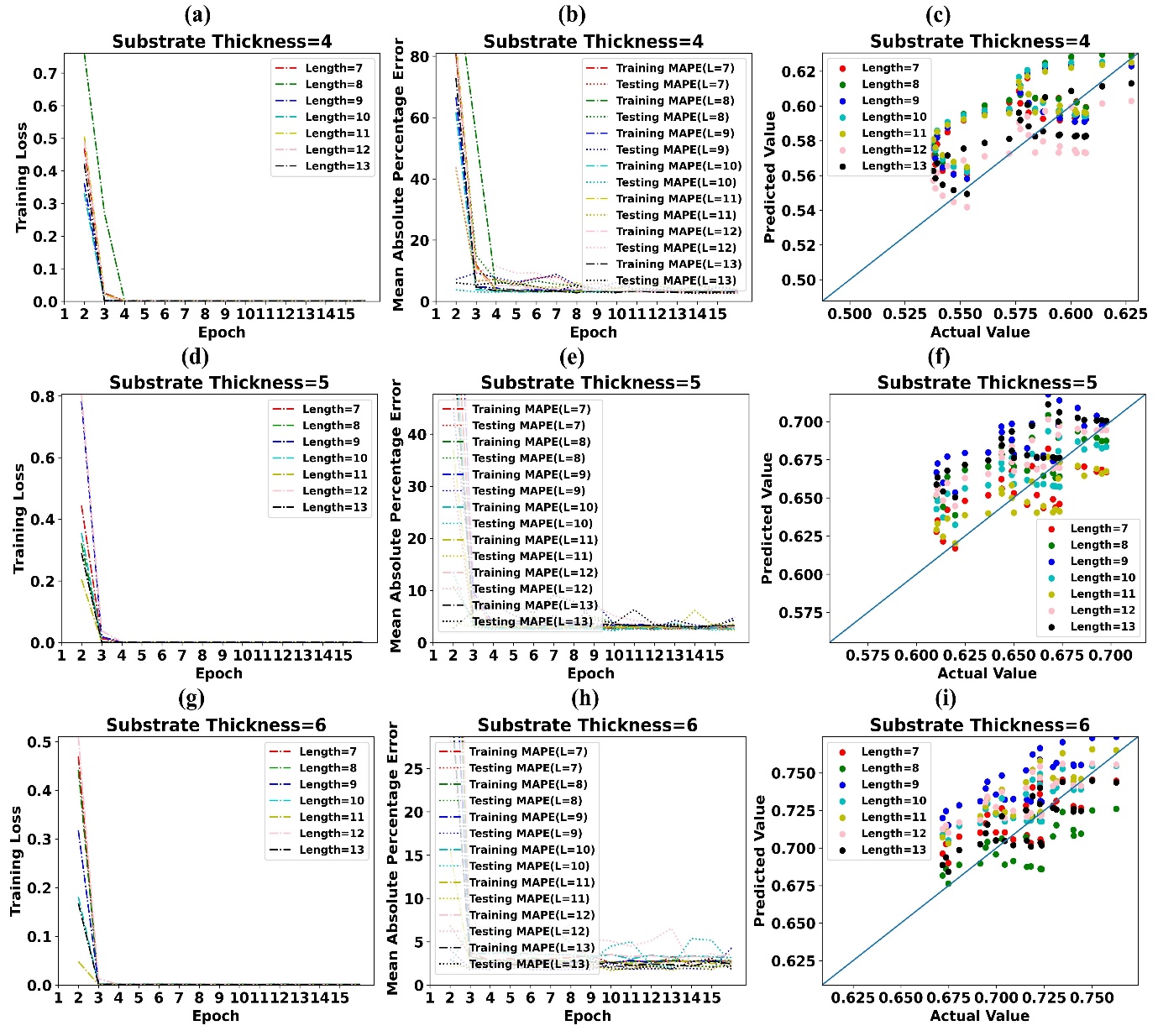


**Fig. S26** (a) Training Loss of LSTM model for Substrate Thickness 4 µm (b) MAPE of LSTM model for Substrate Thickness 4 µm (c) Predicted values of absorption by LSTM vs simulated values of absorption for Substrate Thickness 4 µm (d) Training Loss of LSTM model for Substrate Thickness 5 µm (e) MAPE of LSTM model for Substrate Thickness 5 µm (f) Predicted values of absorption by LSTM vs simulated values of absorption for Substrate Thickness 5 µm (g) Training Loss of LSTM model for Substrate Thickness 6 µm (e) MAPE of LSTM model for Substrate Thickness 6 µm (f) Predicted values of absorption by LSTM vs simulated values of absorption for Substrate Thickness 6 µm
